# Improving Vaccine Coverage Among Older Adults and High-Risk Patients: A Systematic Review and Meta-Analysis of Hospital-Based Strategies

**DOI:** 10.3390/healthcare13141667

**Published:** 2025-07-10

**Authors:** Flavia Pennisi, Stefania Borlini, Rita Cuciniello, Anna Carole D’Amelio, Rosaria Calabretta, Antonio Pinto, Carlo Signorelli

**Affiliations:** 1 Faculty of Medicine, University Vita-Salute San Raffaele, 20132 Milan, Italy; pennisi.flavia@hsr.it (F.P.); borlini.stefania@hsr.it (S.B.); cuciniello.rita@hsr.it (R.C.); damelio.annacarole@hsr.it (A.C.D.); calabretta.rosaria@hsr.it (R.C.); signorelli.carlo@hsr.it (C.S.); 2 PhD National Program in One Health Approaches to Infectious Diseases and Life Science Research, Department of Public Health, Experimental and Forensic Medicine, University of Pavia, 27100 Pavia, Italy; 3 Italian National Immunization Technical Advisory Group (NITAG), 00144 Rome, Italy

**Keywords:** hospital-based vaccination, vaccination coverage, adult immunization, high-risk patients, multi-component interventions, vaccine uptake, systematic review, healthcare interventions

## Abstract

**Background/Objectives:** Adult vaccination remains suboptimal, particularly among older adults and individuals with chronic conditions. Hospitals represent a strategic setting for improving vaccination coverage among these high-risk populations. This systematic review and meta-analysis evaluated hospital-based interventions aimed at enhancing vaccine uptake in adults aged ≥60 years or 18–64 years with at-risk medical conditions. **Methods:** We conducted a systematic review and meta-analysis following PRISMA and MOOSE guidelines. Searches in PubMed, EMBASE, and Scopus identified studies published in the last 10 years evaluating hospital-based interventions reporting vaccination uptake. The risk of bias was assessed using validated tools (NOS, RoB 2, ROBINS-I, QI-MQCS). A meta-analysis was conducted for categories with ≥3 eligible studies reporting pre- and post-intervention vaccination coverage in the same population. **Results:** We included 44 studies. Multi-component strategies (*n* = 21) showed the most consistent results (e.g., pneumococcal uptake from 2.2% to 43.4%, *p* < 0.001). Reminder-based interventions (*n* = 4) achieved influenza coverage increases from 31.0% to 68.0% and a COVID-19 booster uptake boost of +38% after SMS reminders. Educational strategies (*n* = 11) varied in effectiveness, with one study reporting influenza coverage rising from 1.6% to 12.2% (+662.5%, OR 8.86, *p* < 0.01). Standing order protocols increased pneumococcal vaccination from 10% to 60% in high-risk adults. Hospital-based catch-up programs improved DTaP-IPV uptake from 56.2% to 80.8% (*p* < 0.001). For patient education, the pooled OR was 2.11 (95% CI: 1.96–2.27; *p* < 0.001, I^2^ = 97.2%) under a fixed-effects model, and 2.47 (95% CI: 1.53–3.98; *p* < 0.001) under a random-effects model. For multi-component strategies, the OR was 2.39 (95% CI: 2.33–2.44; *p* < 0.001, I^2^ = 98.0%) with fixed effects, and 3.12 (95% CI: 2.49–3.92; *p* < 0.001) with random effects. No publication bias was detected. **Conclusions:** Hospital-based interventions, particularly those using multi-component approaches, effectively improve vaccine coverage in older and high-risk adults. Embedding vaccination into routine hospital care offers a scalable opportunity to reduce disparities and enhance population-level protection. Future policies should prioritize the institutional integration of such strategies to support healthy aging and vaccine equity.

## 1. Introduction

Vaccination stands as a fundamental pillar of preventive medicine, offering one of the most effective and cost-efficient means to reduce the burden of infectious diseases across the lifespan [1,2]. While childhood immunization programs have achieved considerable success, adult vaccination has historically received less focus, resulting in relevant gaps in protection [3]. This is particularly concerning for older adults (aged ≥60 years) and individuals with chronic health conditions or who are immunocompromised. These populations face higher risks of infection and severe complications due to factors such as immunosenescence [4], a high prevalence of multi-morbidity [5], and altered immune responses. Consequently, they are at increased risk for complications from vaccine-preventable diseases, making vaccination not only reduces morbidity and mortality but also prevents disability, loss of autonomy, and functional decline, outcomes particularly relevant in the context of healthy aging [6,7]. Low vaccination coverage in these groups not only leads to increased morbidity and mortality, but also places a significant burden on healthcare systems. Preventable infections among older adults and medically vulnerable individuals often result in prolonged hospital stays, the increased use of intensive care services, antimicrobial resistance, and higher rates of readmission [8,9]. These outcomes not only compromise individual health and functional independence but also generate substantial direct and indirect healthcare costs [10]. Furthermore, unvaccinated individuals may contribute to nosocomial transmission of infectious diseases, posing additional risks to other hospitalized patients and healthcare workers [11]. Improving vaccination coverage in these populations is therefore essential not only for individual protection, but also for enhancing the resilience and sustainability of healthcare delivery systems.

Ongoing demographic transitions further amplify the urgency of optimizing adult immunization strategies. By 2050, the global population of adults aged 65 years and older is expected to exceed 1.5 billion, more than doubling compared to 2019 [12]. At the same time, global life expectancy has increased markedly—from 61.7 years in 1980 to 72.6 years in 2019 and is projected to reach 77.1 years by 2050 [12,13]. As life expectancy rises, so does the prevalence of age-related and chronic diseases, reinforcing the need for effective, scalable preventive interventions such as vaccination. Thus, there is a need for effective prevention strategies to support healthy aging [14].

Despite clear recommendations from health authorities, adult vaccination coverage consistently falls short of public health goals [15,16]. For instance, in the United States, during the 2023–2024 season, only 43.6% of adults aged ≥18 years received the influenza vaccine, with coverage reaching 69.7% among those aged ≥65 years [17]. In the European Union and European Economic Area, the median vaccination coverage among older adults was 45.7% in the 2023–2024 season, down from 59% in 2020–2021, indicating a declining trend [18]. Notably, only a few countries, such as Denmark, have met the 75% coverage target for older adults [18]. In many low-, lower-middle-, and upper-middle-income countries, coverage remains low; for instance, in South Africa, an upper-middle-income country, only 5% of privately insured individuals were vaccinated in 2015 [19]. Pneumococcal vaccine uptake is notably low among at-risk adults under 65 and often inadequate even in those ≥65 years [20]. Similarly, coverage for herpes zoster and needed tetanus boosters is frequently insufficient [20].

The coronavirus disease 2019 (COVID-19) pandemic further strained immunization systems and potentially exacerbated existing challenges and disparities [21,22,23]. Factors contributing to under-vaccination are multi-faceted: limited patient awareness, provider-related factors such as lack of strong recommendations or time constraints, fragmented vaccine delivery systems causing missed opportunities, access barriers including cost and convenience, and vaccine hesitancy often fueled by misinformation [24]. Hospitals emerge as uniquely strategic environments to address these challenges and improve adult immunization coverage [25]. As high-contact settings are frequently utilized by older adults and those with chronic conditions, hospitalization, whether through inpatient admission or emergency department visits, presents an opportunity to identify undervaccinated individuals and provide targeted preventive interventions [25]. Importantly, hospitalized individuals may be more receptive to vaccination due to a heightened awareness of their health status or recent experiences with acute illness [26]. Moreover, hospitals often have access to diagnostic and administrative data, which can support patient identification and tracking of vaccine eligibility and delivery [27]. This context shifts the view of the hospital from solely a site for acute treatment to a vital node within the public health infrastructure capable of delivering crucial preventive care. Leveraging the existing hospital infrastructure, including clinical staff, electronic health records (EHRs), and established patient pathways, can facilitate the integration of vaccination into routine clinical practice. Several systematic and narrative reviews have previously examined interventions to improve adult vaccination coverage [28], often focusing on primary care [3] or community-based settings [29]. However, evidence regarding hospital-based strategies remains limited and fragmented. This fragmentation is largely due to the heterogeneity in study designs (e.g., randomized trials, cohort studies, and quality improvement projects), settings (e.g., inpatient wards, outpatient clinics, and tertiary care centers), and populations (e.g., older adults, immunocompromised individuals, and patients with different chronic diseases). These methodological and contextual differences have limited the generalizability of findings and made comparisons across studies challenging. This systematic review and meta-analysis aims to evaluate the effectiveness of recent hospital-based interventions designed to enhance vaccine coverage among adults aged ≥60 years or between 18 and 64 with one or more high-risk medical conditions. By synthesizing current evidence on various strategies implemented within hospital settings, this report seeks to provide actionable insights to inform clinical and organizational practices, ultimately promoting vaccine equity and improving disease prevention in these vulnerable populations.

## 2. Materials and Methods

This systematic review and meta-analysis was conducted in accordance with the Cochrane Handbook for Systematic Reviews of Interventions and followed the PRISMA 2020 (Preferred Reporting Items for Systematic reviews and Meta-Analyses) and MOOSE (Meta-analysis of Observational Studies in Epidemiology) guidelines for reporting [30]. The study protocol was developed a priori, shared within the research team, and registered in the International Prospective Register of Systematic Reviews (PROSPERO; registration number: CRD420251031996).

### 2.1. Literature Search Strategy

A comprehensive literature search was performed using PubMed/MEDLINE, Embase, and Scopus. The search strategy combined Medical Subject Headings (MeSH) and free-text terms related to hospital settings and vaccination coverage, including: (“hospital*” OR “acute care” OR “inpatient” OR “outpatient”) AND (“vaccination coverage” OR “vaccine uptake” OR “immunization adherence”). The search string used for PubMed is provided in Supplementary Table S1. Search strategies for other databases were adapted based on this string to accommodate the specific syntax and indexing of each database. Boolean operators (AND/OR) were applied to refine the search. The final search was conducted on 13 March 2025. Additional studies were identified by screening the reference lists of included articles and by consulting domain experts.

### 2.2. Inclusion and Exclusion Criteria

Study selection followed the PICOS (Population, Intervention, Comparison, Outcomes and Study) framework (Supplementary Table S3): Population (adults aged ≥60 years or between 18 and 64 with one or more high-risk medical conditions, including oncological diseases, immunosuppressive states, diabetes, cardiovascular or other chronic illnesses), Intervention (hospital-based interventions to improve uptake of recommended vaccines), Comparison (no intervention or usual care), Outcome (vaccine uptake rate), and S (Peer-reviewed experimental and observational studies).

In this review, effectiveness was operationally defined as any measurable change in vaccine uptake rate among eligible adult populations following a hospital-based intervention. Vaccine uptake was expressed as the proportion of eligible individuals who received at least one recommended vaccine dose, either reported as a percentage change (pre-post), odds ratio (OR), relative risk (RR), or absolute difference between control and intervention groups. We included studies with various follow-up periods as long as vaccination uptake was clearly reported post-intervention. This decision reflected the variability in study designs and allowed for the inclusion of both short-term and longer-term evaluations of hospital-based interventions.

Due to heterogeneity across vaccine types and schedules, adherence to complete multi-dose schedules was not required unless explicitly stated in the study. For multi-dose vaccine schedules, we accepted studies that reported the administration of at least the first dose, unless full schedule adherence was specifically evaluated by the study authors. Given the variability in how vaccine completion was reported, we did not require full schedule completion as a criterion for inclusion. When available, information on dose adherence was extracted and reported descriptively.

Eligible studies were peer-reviewed original articles published in English in the last 10 years. To be included, articles needed to be interventions (clinical, behavioral, or structural) to increase any vaccine uptake for adult inpatients and must have measured vaccination uptake as an outcome.

Exclusion criteria included non-original works (e.g., conference abstracts, editorials, books, systematic or narrative reviews, commentaries, expert opinions), ongoing clinical trials, studies not reporting vaccine adherence data specifically for individuals ≥60 years or high-risk patients aged 18–64, studies conducted in non-hospital settings, and publications in languages other than English. Studies published in languages other than English were excluded to ensure accurate comprehension and critical appraisal by all members of the review team. Given the complexity of intervention designs and outcome measures, the ability to thoroughly understand the methodology and interpret the results was prioritized to maintain the quality of data extraction.

### 2.3. Study Selection and Data Extraction

Study selection was performed in two stages: initial screening of titles and abstracts, followed by full-text review of potentially eligible articles. Two reviewers independently assessed all records, resolving disagreements through discussion or consultation with a third senior reviewer. Data extraction was carried out using a predefined and pilot-tested Excel spreadsheet. Extracted variables included: first author, year, study design, country, hospital setting, participants’ characteristics (age, gender, and clinical condition), vaccine type, intervention strategy, sample size, number vaccinated (pre and post intervention, if applicable), and outcome measures. Outcome indicators were reported as described in the original studies, including absolute vaccine uptake rates, percentage changes, and relative measures (when available). Data extraction was conducted in duplicate; discrepancies were resolved by discussion.

### 2.4. Data Synthesis

The study selection process followed PRISMA 2020 guidelines and was documented using a flow diagram. Reasons for exclusion at the full-text stage were recorded. Extracted data were synthesized in tabular form and summarized narratively. Results from statistical analyses were reported using appropriate tables and figures. The included studies were grouped and analyzed based on the type of hospital-based intervention strategy employed to improve vaccine uptake. These categories included: reminders (addressed to patients or staff), education (targeting patients and/or staff), standing order protocols (SOPs), multi-component strategies, clinician prompts, and hospital-based catch-up strategies. In addition, subgroup characteristics such as vaccine type, participant age, and geographic region were summarized to account for clinical and contextual heterogeneity across studies.

### 2.5. Statistical Analysis

A meta-analysis was conducted to evaluate the impact of hospital-based interventions on vaccination uptake by comparing pre- and post-intervention coverage data. Interventions were categorized into seven predefined groups, and a separate meta-analysis was performed for each category that included at least three independent studies. This threshold was met by only two intervention categories, for which meta-analyses were subsequently carried out. For each study, the number of individuals vaccinated before and after the intervention and the corresponding sample sizes were extracted. Only studies with a pre-post design within the same patient population were included; studies using intervention-control or other non-pre/post comparative designs were excluded.

The analysis was performed using ProMeta^®^ 3 (Internovi, Cesena, Italy). The selected effect size was the odds ratio (OR), calculated from binary data (number of vaccinated individuals and total sample size) in two matched groups (pre- and post-intervention). A correlation coefficient of 0.5 between pre- and post-intervention measures was assumed for all included studies, and a sensitivity analysis was performed by repeating the meta-analysis with correlation values of 0.3 and 0.7. The consistency of results across these values supported the robustness of the overall findings.

A second sensitivity analysis was also conducted within each intervention category, limiting the analysis to studies evaluating the same vaccine (e.g., influenza only, pneumococcal only), provided that at least three data points were available for that specific antigen. Meta-analyses were conducted using both fixed-effect and random-effects models, depending on the level of between-study heterogeneity. Heterogeneity was assessed using the I^2^ statistic and interpreted as follows: low (<25%), moderate (25–50%), substantial (50–75%), and considerable (>75%). Publication bias was evaluated through visual inspection of funnel plot asymmetry and formally tested using Egger’s test (*p* < 0.10 considered indicative of potential bias).

### 2.6. Assessment of Study Quality

The risk of bias in the included studies was independently assessed by two reviewers using validated critical appraisal tools, depending on the study design. Discrepancies were resolved by consensus or by a third reviewer when necessary. Observational studies were assessed using an adapted version of the Newcastle–Ottawa Scale (NOS) [31]. The NOS evaluates three domains: selection of participants (0–4 points), comparability of study groups based on control for confounding factors (0–2 points), and outcome or exposure assessment (0–3 points), for a total score ranging from 0 to 9. Studies scoring ≥8 were considered high quality. Non-randomized interventional studies were assessed using the Risk of Bias In Non-randomized Studies of Interventions (ROBINS-I) tool, adapted as appropriate to the context of this review [32]. Based on the ROBINS-I framework, studies were judged as having a low, moderate, serious, or critical risk of bias. Randomized controlled trials (RCTs) were evaluated using the Cochrane Risk of Bias 2 (RoB 2) tool [33]. This tool assesses 5 domains: the randomization process, deviations from intended interventions, missing outcome data, outcome measurement, and selection of the reported result. Each study was categorized as having low risk of bias, some concerns, or high risk of bias. Quality Improvement (QI) studies were identified among the included articles and further appraised using the 16-domain Quality Improvement Minimum Quality Criteria Set (QI-MQCS), specifically developed for critical appraisal of QI studies [34].

## 3. Results

### 3.1. Literature Search

**Figure 1:**
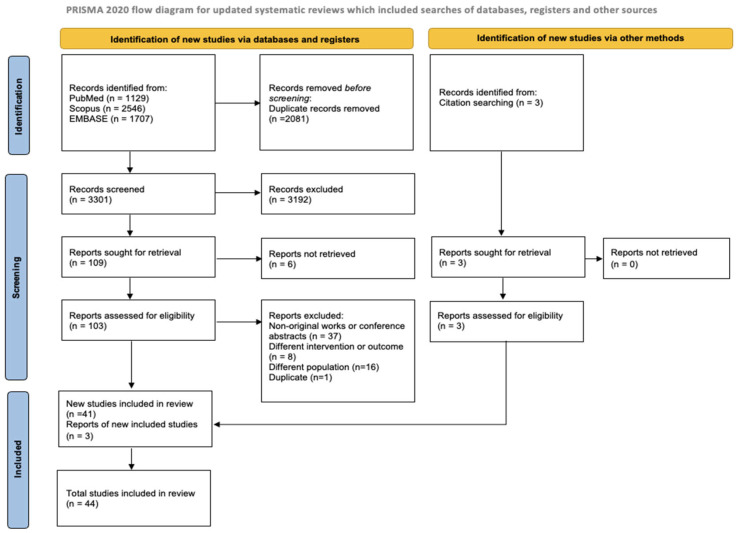
PRISMA study search flow diagram.

A total of 5382 records were identified through database searches in PubMed/MEDLINE (*n* = 1129), Scopus (*n* = 2546), and EMBASE (*n* = 1707). Three [35,36,37] additional articles were included based on reference screening and expert consultation. After removal of duplicates (*n* = 2081), 3301 records were screened based on titles and abstracts. Of these, 109 articles were selected for full-text review. Following full-text assessment, 37 articles were excluded because they were non-original works or conference abstracts, 16 focused on a different population, 1 was a duplicate, 8 investigated a different intervention or outcome, and 6 had no full text available. As a result, 44 articles [35,36,37,38,39,40,41,42,43,44,45,46,47,48,49,50,51,52,53,54,55,56,57,58,59,60,61,62,63,64,65,66,67,68,69,70,71,72,73,74,75,76,77,78] were included in the current systematic review. The study selection process is illustrated in Figure 1.

### 3.2. Descriptive Characteristics of Included Studies

As shown in Table 1, the majority of included studies (*n* = 36) [35,37,38,39,41,42,43,44,45,46,47,48,50,53,54,55,58,60,61,62,63,64,65,66,67,68,69,70,71,72,73,74,75,76,77,78] were conducted in high-income countries, while a smaller number (*n* = 7) [36,49,51,52,56,57,59] originated from upper-middle-income settings, and only 1 [40] from a lower-middle-income country. Country income classification was based on the World Bank 2024–2025 income categories [79]. Taiwan, not individually listed by the World Bank, was considered part of China and thus classified as an upper-middle-income setting.

### 3.3. Geographic Distribution of Studies

**Figure 2:**
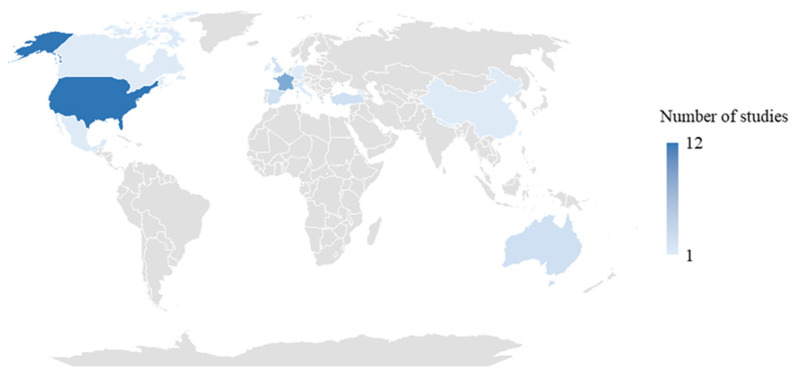
Geographical distribution of included studies.

As illustrated in the geographic map (Figure 2), the included studies were conducted across a diverse range of geographic settings. The majority originated from the United States, 14 studies [35,37,38,41,42,47,53,55,62,65,69,70,71,74], followed by 6 studies in France [39,43,67,68,72,76], 3 studies in Singapore [58,73,78], 2 studies in the United Kingdom [44,66], Turkey [49,56], Spain [50,60], and Australia [54,63]. Other countries represented by a single study included China [52], Germany [48], Mexico [51], Belgium [46], Brazil [36], Nepal [40], Hong Kong [45], Taiwan (China) [57], Ireland [61], Canada [64], Georgia [52], Italy [77], and France and Monaco [75]. This geographic diversity allowed for a broad perspective on vaccination strategies.

### 3.4. Vaccine Subgrouping

Among the studies included in this review, several investigated more than one vaccine. Overall, the most frequently assessed was the influenza vaccine, examined in 25 studies [35,36,37,38,43,44,46,48,50,51,53,55,58,59,60,61,64,67,68,72,73,75,76,77,78], followed by pneumococcal vaccines in 17 studies [37,43,44,45,46,48,49,51,53,55,64,67,68,69,72,75,77], hepatitis B (HBV) in 10 [36,38,43,44,46,48,51,55,62,68], PCV13 in 9 [35,38,41,42,56,63,70,73,74], and PPSV23 in 9 studies [35,38,40,41,42,61,70,73,74]. The herpes zoster (HZ) vaccine was investigated in five studies [35,51,55,71,74] and hepatitis A and COVID-19 vaccines in four studies [47,52,54,66]. Human papillomavirus (HPV) [44,51,55], tetanus, and Tdap [55,68,74] vaccines were each addressed in three studies, while diphtheria–tetanus–pertussis (DTP) [43,67] studies and meningococcal vaccines [44,64] were each assessed in two studies. A single study investigated each of the following: tuberculosis (TB) [38], DTaP-IPV [39], measles-mumps-rubella (MMR) [44], varicella [44], diphtheria [48], pneumonia [36], COVID-19 (booster) [57], Hemophilus influenzae type b (Hib) [64], and pneumococcal (PCV20) [65].

### 3.5. Participant Age Summary

Age data were extracted when available as means or ranges. One study included individuals <40 years [54], three studies [44,46,55] focused on the 40–49 age group, five [36,48,51,68,78] on 50–59, three [43,49,76] on 60–69, and four [39,58,75,77] on 70–79. Additional studies reporting only age thresholds (e.g., ≥65 years) or no age data were excluded from the age-specific summary.

### 3.6. Distribution of Studies Based on Pandemic Period

The majority of included studies (*n* = 32) referred to the pre-pandemic period, five studies [36,38,43,56,67] concerned the pandemic period (some [36,38,56,67] included selected patients from previous years as well) while only seven studies [47,49,52,54,57,59,77] referred to the post-pandemic period. Focusing on the type of vaccine in the pandemic period studies, four studies related to influenza [36,38,43,67] and pneumococcal [38,43,56,67], three related to HBV vaccines [36,38,43], two to DTP [43,67], two to DT [43,67], and one to TB [38]. The types of interventions implemented were multi-component strategies (*n* = 2) [56,67] like EMR alerts, posters, patient/provider education, and dedicated vaccine units, patient education (*n* = 1) [43], patients/staff reminders (*n* = 2) [36,38] like EHR-integrated vaccination checklist for IBD patients at each clinic visit.

Each hospital-based intervention strategy type is presented in a dedicated section below, synthesizing evidence across studies for that specific intervention category, as summarized in Table 2.

### 3.7. Patient/Staff Reminders

Of the included studies, four studies [36,38,41,57], three cohort studies (COHs) [38,41,57], and one randomized controlled trial (RCT) [36] assessed reminder-based strategies aimed at patients and healthcare professionals to improve vaccination coverage. The studies, published between 2019 [41] and 2024 [57], were conducted in the United States [38,41], Brazil [36], and Taiwan [57], and took place in outpatient clinics and veterans’ healthcare facilities. Participants included adults with chronic conditions, hospitalized older adults, and individuals ≥65 years scheduled for COVID-19 booster vaccination. Mean age ranged from 40.64 (13.93) [38] to 72.7 years [41], with female representation between 54% [38] and 96.3% [41]. The interventions involved EHR prompts, discharge instruction note reminders, and personalized SMS messages. The vaccines promoted included influenza, Pneumococcal Conjugate Vaccine 13-valent (PCV13), Pneumococcal Polysaccharide Vaccine 23-valent (PPSV23), hepatitis B, tuberculosis, and COVID-19 boosters. Sample sizes ranged from 139 [36] to 3500 [57]. Post-intervention increases were notable: influenza uptake +119% [38], and COVID-19 booster coverage reached 38% among those who received SMS reminders (compared to a 4% national increase) [57]. Two studies [38,57] reported statistically significant improvements, while two [36,41] others reported statistically significant improvements for only some types of vaccines.

### 3.8. Patient/Staff Education

A total of 11 [43,45,48,49,50,51,59,61,65,72,78] studies investigated educational interventions targeting either patients or healthcare staff. These studies were published between 2015 [45] and 2025 [59] and employed a range of study designs, including six COH [48,49,59,61,72,78], two RCTs [43,45], two [50,65] quasi-experimental studies (QESs), and one [51] cross-sectional study (CSS). The interventions took place in various hospital contexts: four [45,48,51,72] studies were conducted in outpatient clinics, three [43,61,78] in tertiary/university hospitals, and only one [49] had its setting a multicenter or national study, general inpatient wards, specialty program or unit, and veterans’/military facility. Target populations included transplant candidates, older adults with chronic diseases, and immunosuppressed patients. Mean age ranged from 48.5 [72] to 65.15 (±9.2) [59] years, and the percentage of female participants varied widely, from 14.1% [59] to 94.5% [51]. The most frequently targeted vaccines were influenza, pneumococcal, hepatitis B, and Diphtheria, Tetanus, Pertussis (DTP). 

Educational strategies included pre-transplant consultations, structured patient education programs, and information materials. Sample sizes ranged from 39 [43] to 7834 [59] participants. All included studies reported positive changes between pre- and post-intervention periods, with no instances of decreased uptake post-intervention. For example, influenza vaccination coverage increased from 1.6% to 12.1%, corresponding to a relative increase of +662.5% [59], and pneumococcal vaccination uptake rose from 16.1% to 85.7%, with a relative increase of +432.3% [72]. 4 articles [49,59,61,78] report the OR, with OR as high as 9.01 [61] (95% CI 4.40–18.42) for influenza vaccine uptake. Statistical significance was observed in 7 [43,45,49,59,61,72,78] out of 10 studies that reported *p*-values.

### 3.9. Standing Order Protocols (SOPs)

Only one study [74] assessed the implementation of standing order protocols (SOPs), which empower nurses or pharmacists to administer vaccines independently of direct physician orders. The study was conducted in the United States (Minnesota) in 2020 and included one QES design. The setting was limited to outpatient clinics, and participants were adults with chronic health conditions such as heart disease or renal failure. Although detailed demographics were often lacking, interventions led to increases in vaccination rates across multiple antigens. For example, in patients aged 19–64, pneumococcal vaccine coverage rose from 10% to 23% in one site, and in another site improved from 24% to 60%. Relative increases ranged from −14.3% to 2400.0%. Negative values indicate a post-intervention decrease in vaccination uptake compared to baseline, observed in only 2 out of 13 measurements. The study reported statistical significance.

### 3.10. Multi-Component Strategies

Multi-component interventions were the most frequently studied approach, featured in 21 articles [35,37,40,44,47,52,53,56,58,62,63,64,66,67,69,70,71,73,75,76,77] published between 2015 [37] and 2024 [52,56,63,77]. Study designs included 12 QESs [35,37,44,52,53,58,62,63,64,67,70,71], 3 COHs [56,66,69], 3 [40,47,73] quality improvement projects (QIs), and 1 case–control study (CCS) [76], CSS [77], and RCT [75]. Research was conducted across 11 countries, most notably in the USA, the United Kingdom, Singapore, and France. 

Interventions were carried out in various settings, the most representative: seven [47,56,64,66,67,73,77] in a tertiary/university hospital, six [35,37,62,69,70,71] in outpatient clinics, and four [40,53,63,75] in general inpatient wards. Target populations included hospitalized older adults, patients with HIV, and those with chronic illnesses. Age ranged from 48 [44] to 82.4 (±6.9) [63] years, and female representation varied from 8% [58] to 83.5% [35]. The most commonly promoted vaccines were pneumococcal (*n* = 11), influenza (*n* = 9), COVID-19 (*n* = 3), and hepatitis B (*n* = 2). Strategies typically involved combinations of provider education, EHR alerts, discharge checklists, vaccine passports, and patient outreach. Sample sizes ranged from 28 [64] to 29,530 [56] individuals. Out of 21 studies, only 1 [35] reported a negative relative increase, while all others showed positive changes, with the highest reported increase reaching +1872.7% [63]; OR were reported by only 4 [52,58,70,75] studies, while 1 [76] study reported RR. 12 [35,40,44,52,53,56,58,62,63,70,71,76] of 14 studies reported statistically significant results.

### 3.11. Clinician Prompt

Only two studies [42,46] evaluated clinician prompts, typically embedded within the EHR. These included one COH [42] and one RCT [46] conducted between 2017 [43] and 2018 [42] in the USA [42] and Belgium [46]. 

Settings included outpatient clinics and veterans/military facilities. Participants were immunocompromised adults, including HIV-positive veterans and patients with inflammatory bowel disease. The mean age was around 44 years; data on sex distribution were around 47% female. Vaccines promoted included pneumococcal, PCV13, PPSV23, influenza, hepatitis B, and tetanus. Interventions involved automatic alerts to providers, supplemented by reminder letters to patients. Sample sizes ranged from 99 [42] to 505 [46]. One study [42] showed a significant increase in pneumococcal vaccination from 0% to 38.4% within 180 days. In the second study, significant differences in vaccination uptake were observed between the control and intervention groups: influenza (10% vs. 36%), pneumococcal (23% vs. 62%), hepatitis B (5% vs. 27%), and tetanus (2% vs. 33%).

### 3.12. Hospital-Based Catch-Up Strategy

A total of five studies [39,54,55,60,68] implemented catch-up vaccination strategies during hospitalization, aimed at patients with incomplete vaccine histories. These studies, conducted from 2020 [39] to 2023 [54], included three cohort studies [54,55,68] and two [39,60] randomized trials across four countries. Participants included elderly inpatients and individuals with severe mental illness or chronic gastrointestinal conditions. Mean age ranged from 36.5 [54] to 81.4 [39] years, and female representation ranged between 36% [68] and 65.8% [39]. Vaccines addressed included influenza, pneumococcal, and hepatitis A/B. Interventions typically included real-time eligibility assessments and vaccine offers during hospitalization. Sample sizes ranged from 142 [54] to 524 [60]. One study [39] reported a relative increase of 43.8% in the intervention group (vs. 6.3% in the control group). One study [55] stood out by reporting predominantly negative relative changes across several vaccines, including –29.5% for influenza, –5.0% for pneumococcal, –34.0% for HZ, –37.8% for HAV, and –8.7% for HBV, with only modest increases for Tdap (+2.1%) and HPV (+34.9%). This contrasts with the overall trend of positive post-intervention changes observed in the majority of studies. One study reported OR up to 2.48 [60] (95% CI 1.6–3.8).

### 3.13. Meta-Analysis

In our meta-analysis, both patient education interventions and multi-component strategies showed substantial effectiveness across different vaccine types, despite the high heterogeneity observed among the included studies.

**Figure 3:**
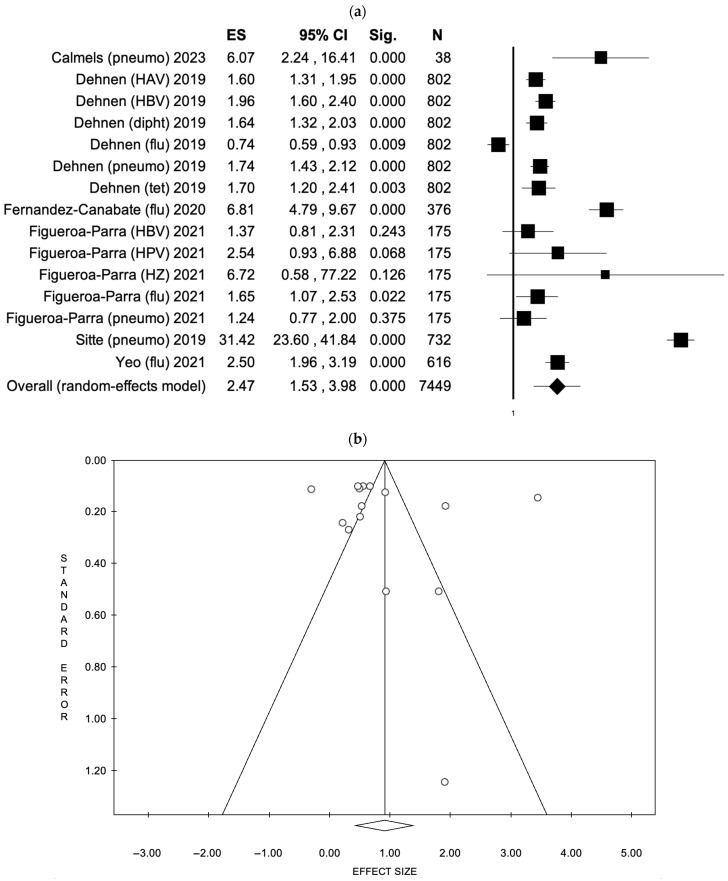
(**a**) Forest plot and (**b**) funnel plot of the random effect model assessing patient education interventions [43,48,50,51,72,78].

For patient education interventions, six studies [43,48,50,51,72,78] encompassing 15 vaccine uptake outcomes were analyzed. The fixed effects model (FEM) yielded an effect size (ES) of 2.11 (95% CI: 1.96–2.27, *p* < 0.001) based on a total sample of 7449 participants, although significant heterogeneity was detected (I^2^ = 97.21%, *p* < 0.001). When using the random effects model (REM), the ES increased to 2.47 (95% CI: 1.53–3.98, *p* < 0.001). There was no indication of publication bias, as confirmed by the funnel plot and Egger’s test (intercept: 2.31, *p* = 0.524). These results are presented in Figure 3 (a: Forest plot; b: Funnel plot) and S4 (a: Forest plot; b: Funnel plot).

**Figure 4:**
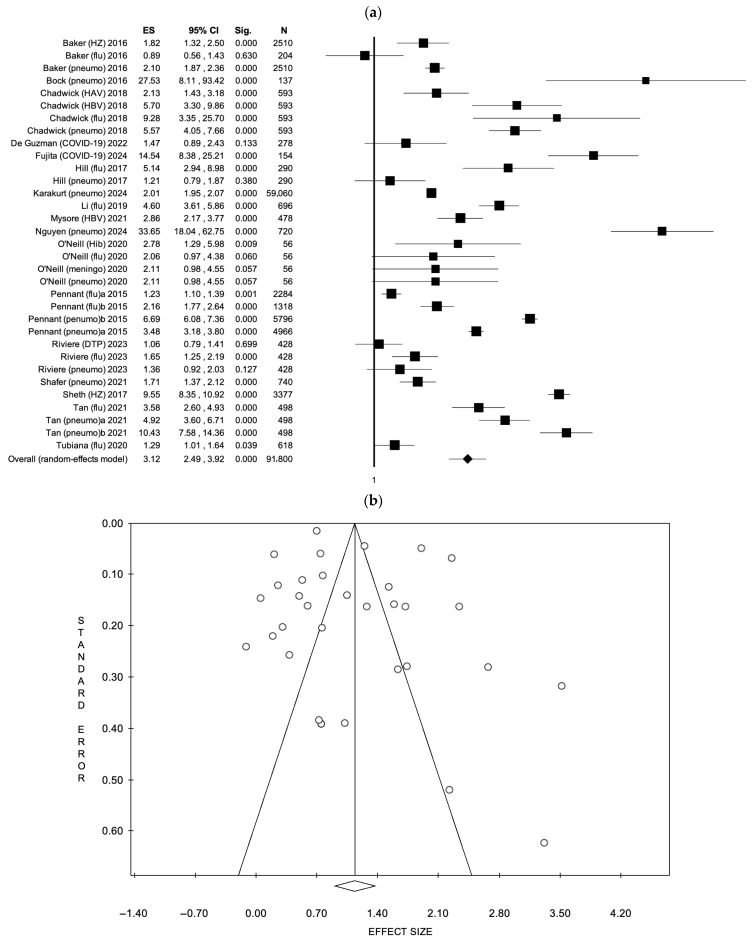
(**a**) Forest plot and (**b**) funnel plot of the random effect model assessing multi-component strategies [35,37,40,44,47,52,53,56,58,62,63,64,67,69,71,73,76].

For multi-component strategies, 17 studies [35,37,40,44,47,52,53,56,58,62,63,64,67,69,71,73,76] comprising 33 vaccine uptake outcomes were evaluated. The FEM reported an ES of 2.39 (95% CI: 2.33–2.44, *p* < 0.001), with considerable heterogeneity (I^2^ = 98.01%, *p* < 0.001), based on a total of 91,800 participants. Under the REM, the ES increased to 3.12 (95% CI: 2.49–3.92, *p* < 0.001). As with the previous analysis, no evidence of publication bias was found (Egger’s test intercept: 2.36, *p* = 0.133). The results are illustrated in Figure 4 (a: forest plot; b: funnel plot) and S5(a: forest plot; b: funnel plot).

### 3.14. Sensitivity Analysis

To assess the robustness of the assumed correlation between pre- and post-intervention data, a sensitivity analysis was conducted using correlation coefficients of 0.3 and 0.7 in addition to the primary value of 0.5. The results across all models remained consistent, with no meaningful variation in the direction or magnitude of the effect sizes. Given the comparability of outcomes across these assumptions, only the estimates based on the 0.5 correlation coefficient are reported in the main analysis.

**Figure 5:**
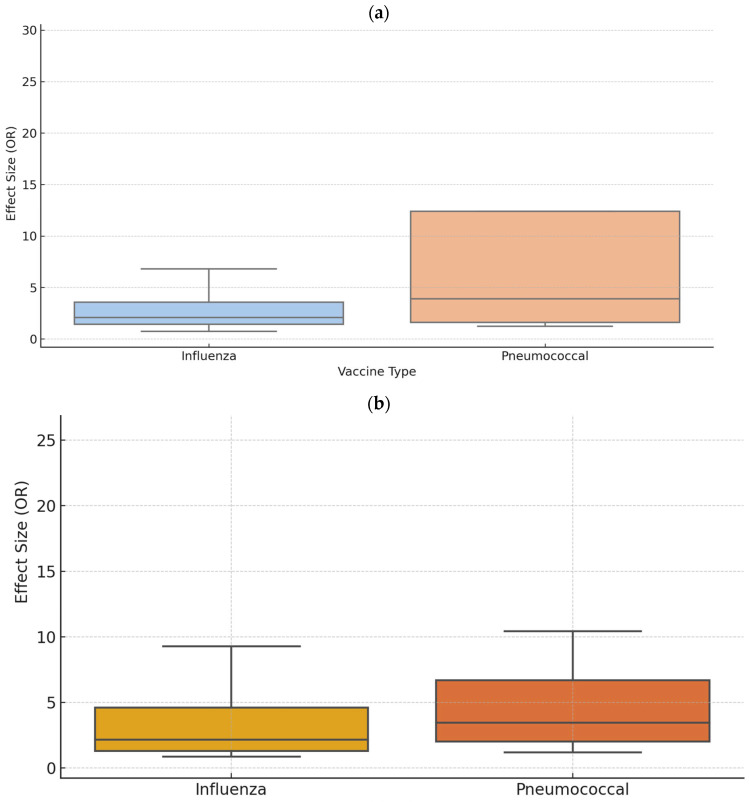
Distribution of effect sizes (odds ratios) for (**a**) patient education and (**b**) multi-component hospital-based strategies by vaccine type: sensitivity analysis comparing influenza and pneumococcal vaccines.

We conducted a sensitivity analysis stratified by vaccine type. For patient education interventions, analysis restricted to studies using the influenza vaccine included 4 studies [48,50,51,78] with a total of 1969 participants. The effect sizes (ORs) ranged from 0.74 to 6.81 across the studies (Figure 5a). The FEM yielded an ES of 1.74 (95% CI: 1.51–2.00, *p* < 0.001), although heterogeneity remained high (I^2^ = 97.55%, *p* < 0.001). The REM indicated an ES of 2.13 (95% CI: 0.83–5.43, *p* = 0.114). For pneumococcal vaccines, four studies [43,48,51,72] with a combined sample of 732 participants showed an ES of 3.93 (95% CI: 3.37–4.58, *p* < 0.001) under the FEM. The REM reported an ES of 4.49 (95% CI: 0.78–25.89, *p* = 0.092). Pneumococcal vaccination showed a wider range of ORs (1.24 to 31.42), driven by a single outlier study, with a visually higher median (Figure 5a).

Regarding multi-component strategies, eight studies [35,37,44,53,58,64,73,76] focused on influenza vaccination (*n* = 6557) reported an ES of 1.76 (95% CI: 1.62–1.91, *p* < 0.001) under the FEM, with significant heterogeneity (I^2^ = 94.87%, *p* < 0.001). The REM indicated an ES of 2.42 (95% CI: 1.59–3.69, *p* < 0.001). For studies using pneumococcal vaccines (11 studies, *n* = 76,292) [35,37,40,44,53,56,63,64,67,69,73], the FEM produced an ES of 2.35 (95% CI: 2.29–2.41, *p* < 0.001), with pronounced heterogeneity (I^2^ = 98.65%, *p* < 0.001). The REM showed a higher ES of 3.91 (95% CI: 2.77–5.53, *p* < 0.001). Influenza-related studies showed a more compact distribution (median 2.2), while pneumococcal studies demonstrated a broader range and higher variability, including outlier values exceeding OR > 25 (Figure 5b).

### 3.15. Quality Assessment

The quality assessment of the 44 studies was evaluated using four different checklists, based on the type of study:

- The NOS checklist was used to qualitatively assess the risk of bias in 17 [38,41,42,48,49,54,55,56,57,59,66,68,69,72,76,77,78] of the studies included in the review, all of which were observational studies. Among the 17 articles where it was applied, 14 [38,41,42,54,55,56,57,59,66,68,69,72,77,78] scored ≥ 8/9 points, 3 [48,49,54] received a total score of ≤ 7/9 (Supplementary Table S4).
- The QI-MQCS was applied to 12 articles [35,37,40,47,51,58,61,62,64,70,71,73]. Among them, 10 [37,40,51,58,61,62,64,70,71,73] scored ≥ 14/16, whereas 2 articles [35,47] received a score of ≤ 13/16 (Supplementary Table S5).
- The RoB 2 tool was used to qualitatively assess the risk of bias in 7 [36,39,43,45,46,60,75] of the studies included in this review. Three studies had low risk of bias [43,45,60], two showed some concerns [39,75], and two were rated as high risk of bias [36,46] (Supplementary Table S6).
- Among the eight [44,50,52,53,63,65,67,74] non-randomized studies assessed with ROBINS-I, three had a moderate risk of bias [50,52,63], four had a serious risk [44,53,67,74], and one had a critical risk of bias [65] (Supplementary Table S7).

In Table 3, all studies were reported with the specific checklist used and the score was associated with a color that graphically represents the risk of bias. Overall, 27 studies [37,38,40,41,42,43,45,51,55,56,57,58,59,60,61,62,64,66,68,69,70,71,72,73,76,77,78] had a low risk of bias, 3 had a moderate risk of bias [50,52,63], 4 had a serious risk of bias, 8 studies had a high risk of bias [35,36,46,47,48,49,54,65], and 2 studies [39,75] had some concerns.

## 4. Discussion

The findings from this systematic review with meta-analysis indicate that, as a whole, hospital-based interventions can significantly improve vaccination uptake among older adults and high-risk populations. Across the 44 included studies, a wide range of strategies, such as patient and staff education, electronic reminders, standing order protocols, and multi-component interventions, were associated with increased coverage for vaccines, including influenza, pneumococcal, hepatitis B, tetanus, and COVID-19. However, the review also found that the effectiveness of interventions varied considerably. While most studies reported positive changes in uptake, particularly those lacking control groups, showed minimal or even negative changes. Notably, several studies reported absolute increases in uptake exceeding 30%. Hospital-based interventions, if adequately structured and integrated, can address several barriers that typically hinder vaccine uptake in community settings.

This meta-analysis also provided quantitative evidence supporting the efficacy of both patient education interventions and multi-component strategies in enhancing vaccine uptake across diverse populations and vaccine types. Notably, multi-component interventions exhibited greater pooled effect sizes compared to education-only strategies, suggesting a potential cumulative or synergistic effect when multiple implementation components are deployed concurrently.

Patient education interventions were associated with significant improvements in vaccine adherence, particularly in the context of pneumococcal vaccination. However, the high heterogeneity observed (I^2^ > 97%) and the broad confidence intervals under the random effects model underscore the methodological and contextual variability across studies. The limited precision in effect size estimates, especially for influenza-focused education interventions, may reflect variability in intervention design, delivery modalities, population literacy levels, and baseline vaccine hesitancy.

Multi-component strategies yielded higher and more consistent effect sizes, with a particularly notable impact observed in studies targeting pneumococcal vaccination. These findings align with implementation science literature, which emphasizes that interventions addressing multiple barriers, such as structural access, provider recommendation, behavioral prompts, and informational deficits, are more likely to produce meaningful changes in preventive health behaviors. The significantly higher ES observed in the REM further supports the robustness of these approaches, despite substantial between-study variability (I^2^ > 98%).

These results align with findings from other systematic reviews on interventions in primary care [80,81] and community settings [82], which demonstrate that multifactorial approaches are the most effective. However, our review highlights the added value of the hospital setting, where the integration of organizational and technological strategies enables reaching high-risk populations often excluded from traditional vaccination pathways.

This review provides an important and timely contribution to the existing literature by offering an up-to-date synthesis of hospital-based strategies to improve adult vaccination uptake, including during the COVID-19 era. To our knowledge, this is the first systematic review and meta-analysis to assess hospital-based interventions for adult vaccination that includes COVID-19 vaccines. Several studies addressed COVID-19 immunization using strategies such as bedside catch-up programs, SMS reminders, and multi-component campaigns. For instance, Fujita et al. [52] implemented bedside COVID-19 vaccination for hospitalized adults, leading to an increase in vaccination coverage from 21.0% to 79.0%, with a significant post-intervention odds ratio of 3.92 (95% CI: 2.24–6.87, *p* < 0.001). Similarly, Hooper et al. [54] reported that offering COVID-19 vaccines to psychiatric inpatients resulted in 28.3% of eligible individuals being vaccinated during hospitalization, a marked improvement compared to general population rates at the time. Lee et al. [57] demonstrated the effectiveness of SMS-based reminders: a 38% relative increase in booster uptake was observed following message delivery, significantly outperforming the national average increase of only 4%. Finally, De Guzman et al. [47] showed that even a relatively simple intervention, such as a multi-phase campaign using inpatient pamphlets and staff alerts, resulted in a 43.9% relative increase in inpatient COVID-19 vaccine coverage.

However, during the COVID period, fear and anxiety about the coronavirus may have also played a role in the results and delays in some vaccinations. Calmels et al. [43] underlined that for several patients, the vaccinations recommended were delayed due to the COVID vaccination campaign. For the same reasons, since vaccines could only be given 2 weeks after a SARS-CoV2 vaccination, not all of them were injected on the day of the consultation. Furthermore, Riviere et al. [67] stated that delaying the training in the third center could have modified the reassessment of seasonal influenza VC because the influenza epidemic and the national vaccination campaign would have been completed long before the evaluation.

Among the hospital-based strategies analyzed, multi-component interventions consistently emerged as the most effective and scalable approach to improving vaccine uptake in high-risk adult populations. These interventions, which combine several elements, such as staff education, EHR prompts, structured discharge planning, and patient-targeted communication, were not only the most frequently implemented (*n* = 21) [34,37,41,44,49,51,54,56,60,61,62,64,65,66,68,69,70,72,74,75,76], but also yielded the largest and most consistent improvements in coverage across diverse settings and vaccine types. For instance, a comprehensive pneumococcal campaign conducted in Turkey [56] led to a 74.4% relative increase in vaccination coverage (from 15.0% to 26.2%, *p* < 0.001), while a bundled intervention in Singapore [73] raised influenza uptake from 63.0% to 86.0% (+36.5%). Studies using multi-faceted models also reported broader impacts, such as fewer vaccine refusals, improved documentation, and higher clinician engagement—underscoring their systemic value. These results are consistent with previous studies [82,83] focused on different populations. A recent systematic review and meta-analysis [84] found that multi-component interventions generally exhibited excellent effectiveness in vaccination (54.3% increase, 95% CI: 40.5 to 69.6%), with the combination of dialog, incentive and reminder/recall proving more effective than other multi-component interventions.

Educational interventions have traditionally been the most widely used approach to improve vaccination rates; nonetheless, their actual effectiveness remains controversial [83,85]. In our study, patient and provider education, though widely employed, demonstrated mixed results. While some studies showed substantial improvements, such as a 232.9% increase in influenza vaccination among kidney transplant candidates following intensive counseling [43], others yielded minimal or statistically non-significant changes [50,65]. This variability may reflect differences in the format, duration, and intensity of educational content. Previous evidence suggests that education alone is insufficient to drive behavior change in complex health systems. A review [86] of educational interventions for adult vaccination concluded that standalone education modestly improves knowledge (10% relative increase) but rarely translates into meaningful uptake unless combined with active follow-up or vaccine access. Dubé et al. [85] conducted a systematic review of 15 prior reviews and concluded that the evidence supporting educational interventions was generally limited and inconsistent. Similarly, a comprehensive review published in 2000 [87] found that reminder-based strategies targeting either healthcare providers or patients were effective in improving vaccination coverage, whereas education-only interventions did not yield significant benefits. These findings suggest that while education on vaccine safety [88] and benefits is essential, it is rarely sufficient alone to meaningfully increase vaccine uptake. Education remains essential but should be embedded within broader structural or behavioral frameworks. Further research is warranted to clarify which patient populations are most likely to benefit from traditional educational approaches, such as those already inclined to accept vaccination, and in which groups, such as vaccine-hesitant individuals, such interventions may be less effective or even counterproductive.

Reminder-based strategies (e.g., EHR alerts, SMS messages) were effective, particularly when personalized and timely. Prior literature confirms these effects, showing that patient reminders significantly increase vaccination rates, particularly for adult influenza, with a reported RR of 1.29 (95% CI: 1.17–1.43) [89]. Notably, reminders perform best when they target both patients and providers and are coupled with real-time decision support.

SOPs empower nurses and pharmacists to administer vaccines without direct physician orders, streamlining workflows and reducing missed opportunities. In our review, SOPS produced modest increases (e.g., PPSV23 from 24% to 60% in one site), yet these gains are consistent with previous evidence. By standardizing and streamlining vaccine administration, SOPs help integrate vaccination into routine care processes in outpatient settings, reducing dependence on individual clinician orders and thereby minimizing missed opportunities. Their effectiveness appears greatest when supported by staff training, leadership buy-in, and integration with EHR systems [90,91,92].

Catch-up interventions during hospitalization or follow-up visits demonstrated high potential, especially for patients with complex needs or limited outpatient access. In one included study, DTaP-IPV uptake rose from 56.2% to 80.8% among hospitalized older adults (*p* < 0.001 [39]. This is in line with the “opportunistic immunization” in acute care settings to close protection gaps [93]. However, practical barriers, including vaccine availability, staff workload, and competing clinical priorities, must be addressed to ensure effectiveness.

### 4.1. Organizational and Public Health Implications

Organizationally, incorporating vaccination into routine hospital care demands careful planning but is feasible. Common implementation barriers include a lack of standardized procedures, insufficient training, physician shortages [94], and workflow disruptions [95]. These can be mitigated through well-defined SOPS, integration into electronic systems, and assigning responsibility to dedicated personnel (e.g., vaccination nurses or pharmacists). Involving staff early in the design process improves ownership and acceptability. Several studies underscore the importance of a multidisciplinary approach, engaging clinicians, nurses, pharmacists, and IT personnel to ensure smooth operationalization.

Implementing standardized protocols, electronic reminders, and catch-up vaccination programs should be considered a priority in hospital policies, as these strategies have the potential to reduce health disparities and substantially improve vaccination coverage among the most vulnerable adults.

From a public health perspective, expanding vaccination beyond traditional outpatient settings is essential to increase coverage and equity. Reaching broader segments of the population requires bringing vaccines closer to where people already receive care or services. In this context, hospitals, as well as schools [96] and community pharmacies [97], play a strategic role in capturing individuals who may otherwise remain unvaccinated due to logistical, socioeconomic, or informational barriers. Offering vaccines during hospital stays is particularly impactful for older adults and people with chronic conditions.

Sustained implementation of hospital-based vaccination initiatives can yield long-term benefits, including reduced morbidity, fewer readmissions, and improved patient awareness. Ensuring that patients are discharged with updated immunization records and clear follow-up instructions supports continued coverage. On a systems level, scaling these models can significantly improve national vaccination coverage, particularly among high-priority groups.

Policymakers and public health authorities play a crucial role in sustaining and scaling these interventions by embedding them into national immunization plans, establishing supportive legislation, and aligning hospital incentives with vaccination targets. Frontline practitioners, including nurses, primary care physicians, and pharmacists, are equally vital for translating policy into action and ensuring the continuity of care after discharge. Collaborative engagement between policy and practice is essential to optimize uptake and reduce structural barriers.

In summary, these findings reinforce the value of educational and multi-component interventions as effective strategies to promote vaccine uptake. Patient education alone can yield significant benefits, particularly when adapted to specific sociodemographic and clinical contexts. However, the greater efficacy observed with multi-component approaches highlights the importance of integrated, system-level strategies in vaccination promotion. Future research should aim to elucidate the optimal combination and sequencing of intervention components, with particular attention to cost-effectiveness, scalability, and contextual adaptability in real-world public health settings.

### 4.2. Limitations

This systematic review and meta-analysis has several limitations. First, a substantial proportion of included studies were observational and thus subject to selection bias, confounding, and lack of randomization. Although the risk of bias was systematically assessed using validated tools, the overall methodological quality varied, with some studies demonstrating moderate to serious risk of bias. Publication bias cannot be ruled out, as studies with positive findings are more likely to be published than those reporting null or negative results. In addition, the exclusion of the gray literature (such as technical reports, theses, or conference proceedings not published in peer-reviewed journals) may have further amplified this bias, thereby reducing the overall representativeness of the available evidence.

Most interventions were conducted in high-income countries, potentially limiting applicability to low- and middle-income settings. Furthermore, variations in vaccine types and reporting metrics (e.g., absolute vs. relative increases) complicate cross-study comparisons.

Moreover, only peer-reviewed articles published in English were included. The restriction to English-language publications was applied to ensure accurate comprehension and critical appraisal of the studies by all members of the review team, given the methodological complexity of the interventions assessed. However, this choice may have introduced language bias and potentially excluded relevant data from non-English-speaking countries. Future updates may consider including non-English studies and the gray literature sources, provided appropriate translation and quality appraisal mechanisms are available.

Data extraction was conducted using standardized protocols and independently cross-checked by multiple reviewers; however, extraction errors cannot be entirely ruled out, particularly in cases of ambiguous or incomplete reporting. The future use of automated tools may help enhance the accuracy and consistency of data extraction in such contexts.

Among the various intervention categories identified, meta-analysis was possible only for patient education and multi-component strategies. This was primarily due to the limited number of studies per category, missing data (e.g., total sample size or baseline adherence), or substantial heterogeneity in study design. As a result, the generalizability of our findings to other intervention types remains limited. Further standardized studies are needed to enable broader quantitative synthesis.

Further research is needed to assess the long-term sustainability and cost-effectiveness of these interventions, as well as their impact on specific subgroups, such as individuals with multiple comorbidities or those from socioeconomically disadvantaged backgrounds. In addition, studies evaluating the scalability of these approaches in low- and middle-income countries would be valuable to inform global policy.

## 5. Conclusions

Hospital-based interventions represent an effective and underutilized opportunity to increase adult vaccination coverage, particularly among older adults and high-risk populations. The meta-analysis demonstrated that multi-component strategies and structured patient education interventions are effective in significantly increasing vaccine uptake. Hospitals offer a critical point of contact to reach individuals who may otherwise face structural, informational, or behavioral barriers to vaccination. In the context of an aging population and ongoing risks from vaccine-preventable diseases, institutionalizing adult vaccination within hospital settings is both evidence-based and important for advancing immunization equity and public health resilience.

## Figures and Tables

**Table 1:** Characteristics of the included studies.

First Author, Year	Study Design	Country	Income	Setting	Participants Description	Intervention Description	Vaccine	Type of Strategy
Baker D.W., 2016 [35]	QES	USA	H	Outpatient Clinics	Adults with RA	Electronic reminders with order sets, physician audit/feedback, patient outreach, and optional printed prescription for HZ vaccination	Influenza, PCV13, PPSV23, HZ	Multi-component strategies
Bernasko N., 2023 [38]	COH	USA (Pennsylvania)	H	Outpatient Clinics	Adults ≥18 with IBD on immunosuppressive therapy	EHR-integrated vaccination checklist for IBD patients at each clinic visit	Influenza, PCV13, PPSV23, HBV, TB	Patients/Staff Reminders
Blanchi S., 2020 [39]	RCT	France	H	General Inpatient Wards	Hospitalized adults ≥65	In-hospital DTaP-IPV vaccination offered to eligible patients; control received verbal information only	DTaP-IPV	Hospital-based catch-up strategy
Bock A., 2016 [40]	QI	Nepal	L-M	General Inpatient Wards	Adults ≥65 or adults 19–64 with chronic disease, active smoking, or immunosuppression	Staff education combined with standing orders and a discharge checklist authorizing nurse vaccination	PPSV23	Multi-component strategies
Burka A., 2019 [41]	COH	USA (Tennessee)	H	Veterans/Military Facilities	Hospitalized veterans ≥65	Discharge instructions included EMR-based patient-specific vaccine guidance per ACIP recommendations	PCV13, PPSV23	Patients/Staff Reminders
Burns C., 2018 [42]	COH	USA (Ohio)	H	Veterans/Military Facilities	HIV-positive veterans not current with PCV13 or PPSV23, with at least one clinic visit in prior 2 years	Virtual clinic used EMR alerts and mailed reminders to notify providers and patients about pneumococcal vaccines	PCV13, PPSV23	Clinician prompt
Calmels A., 2023 [43]	RCT	France	H	Tertiary/University Hospital	Adults ≥18 awaiting kidney transplantation, randomized into standard or reinforced vaccination consultation groups	Pre-transplant patients received either ID consultation (Intervention) or a written vaccine recommendation (Control)	Influenza, Pneumococcal, HBV, DTP	Patient education
Chadwick D., 2018 [44]	QES	United Kingdom	H	Specialty Programs or Units	Adults PLWH attending HIV clinic for vaccination	Use of a patient-held vaccine passport documenting recommended immunizations and follow-up instructions	HAV, HBV, Pneumococcal, Influenza, HPV, MMR, Varicella, Meningococcal	Multi-component strategies
Chan S., 2015 [45]	RCT	Hong Kong	H	Outpatient Clinics	Adults ≥65 with chronic diseases	Brief nurse-led dual-format education (3 min phone + 3 min in-person) before/during appointments	Pneumococcal	Patient education
Coenen S., 2017 [46]	RCT	Belgium	H	Outpatient Clinics	Adults ≥18 with IBD	15 min guideline-based education session provided by IBD nurse during 1-on-1 consultation	Influenza, Pneumococcal, HBV, Tetanus	Clinician prompt
De Guzman E., 2022 [47]	QI	USA (Washington D.C.)	H	Tertiary/University Hospital	Hospitalized adults on the medical service	Multi-phase educational campaign with staff email alerts, hospital posters, and inpatient vaccine pamphlets	COVID-19	Multi-component strategies
Dehnen D., 2019 [48]	COH	Germany	H	Outpatient Clinics	Liver transplant adults	Telephone reminders by LT outpatient clinic requesting patients to bring vaccination documents to next visit	Tetanus, Diphtheria, HAV, HBV, Pneumococcal, Influenza	Patient education
Ekin T., 2023 [49]	COH	Turkey	U-M	Multicenter or National Studies	Adults ≥18 with cardiovascular risk factors	Self-administered questionnaire and physician-delivered pneumococcal vaccine education	Pneumococcal	Patient education
Fernández-Cañabate E., 2020 [50]	QES	Spain	H	General Inpatient Wards	Adults ≥18 who were receiving BT or who had started pre-BT testing	Nurse-led education on vaccine benefits provided to patients starting or receiving BT	Influenza	Patient education
Figueroa-Parra G., 2021 [51]	CSS	Mexico	U-M	Outpatient Clinics	Rheumatic disease adults	Distribution of vaccination manual, patient charts, vaccination passes, and barrier-assessment tools for rheumatic patients	Influenza, Pneumococcal, HZ, HPV, HBV	Patient education
Fujita A., 2024 [52]	QES	Georgia	U-M	Veterans/Military Facilities	Adults hospitalized in medical, surgical, and psychiatric wards	Bedside COVID-19 vaccination delivered to eligible inpatients by trained staff after screening by healthcare personnel	COVID-19	Multi-component strategies
Guerra G.L., 2023 [36]	RCT	Brazil	U-M	Outpatient Clinics	Adults with diabetes mellitus	NA	Pneumonia, HBV, Tetanus, Influenza	Patients/Staff Reminders
Hill J.D., 2017 [53]	QES	USA (Kansas City)	H	General Inpatient Wards	Inpatients hospitalized for cardiovascular care	Pharmacy techs reviewed charts, alerted nurses for follow-up via phone or in person	Influenza, Pneumococcal	Multi-component strategies
Hooper K., 2023 [54]	COH	Australia	H	Specialty Programs or Units	Adults with severe mental illness admitted to the Mental Health Unit	COVID-19 vaccines offered to eligible psychiatric inpatients during hospitalization through structured screening and referral	COVID-19	Hospital-based catch-up strategy
Hussain N., 2021 [55]	COH	USA (Connecticut)	H	Outpatient Clinics	Adults with IBDs attending ≥2 clinic visits	Immediate post-visit vaccination by IBD nurse vs. referral to PCP/pharmacy; same EMR checklist used in both clinics	Influenza, Pneumococcal, HZ, HAV, HBV, Tdap, HPV	Hospital-based catch-up strategy
Karakurt Z., 2024 [56]	COH	Turkey	U-M	Tertiary/University Hospital	Adults ≥65 or with chronic diseases	Multi-channel reminder campaign including EMR alerts, posters, patient/provider education, and dedicated vaccine units	PCV13	Multi-component strategies
Lee Y., 2024 [57]	COH	Taiwan (China)	U-M	Veterans/Military Facilities	Adults ≥65, already completed primary cycle anti-COVID, with outpatient appointment booked between April and May 2022	Personalized SMS reminders with booster booking links sent 1 week before scheduled visit	COVID-19 (booster)	Patients/Staff Reminders
Li A., 2019 [58]	QES	Singapore	H	Specialty Programs or Units	Adults with COPD, in outpatient follow-up	3-part intervention: educational posters, physician briefings with visual reminders, nurse alerts, and new EHR system	Influenza	Multi-component strategies
Liu C., 2025 [59]	COH	China	U-M	Specialty Programs or Units	Adults with COPD enrolled in a real-world follow-up study	Intensive education for COPD patients: in-person counseling, brochures, and SMS reminders 3 months after visit	Influenza	Patient education
Muñoz-Miralles R., 2022 [60]	RCT	Spain	H	Multicenter or National Studies	Adults ≥60, healthy or with conditions, and adults <60 with risk factors for influenza complications	Tailored verbal and written intervention by providers addressing specific reasons for influenza vaccine refusal	Influenza	Hospital-based catch-up strategy
Murray K., 2020 [61]	COH	Ireland	H	Tertiary/University Hospital	Adults receiving immunosuppressive therapy for rheumatologic diseases	Education sessions for rheumatology staff and integration of vaccination prompts into routine workflow using point-of-care tools	PPSV23, Influenza	Staff education
Mysore P., 2021 [62]	QES	USA (New England)	H	Outpatient Clinics	Adults with stage 4–5 chronic kidney disease, unvaccinated or non-immune to HBV, with ≥2 clinic visits in the past 2 years	EHR-based CKD registry with reminders, co-located nurse visits, and provider awareness campaign for vaccination	HBV	Multi-component strategies
Nguyen T., 2024 [63]	QES	Australia	H	General Inpatient Wards	Adults ≥70	Pharmacist audit of inpatients with physician alerts; standardized EMR-based communication and vaccine tracking	PCV13	Multi-component strategies
O’Neill N., 2020 [64]	QES	Canada	H	Tertiary/University Hospital	Relapsed or refractory ITP or TTP adults	Asplenia toolkit developed, implemented, and evaluated for effectiveness and user satisfaction	Pneumococcal, Hib, Meningococcal, Influenza	Multi-component strategies
Pacheco C., 2024 [65]	QES	USA (Texas)	H	Veterans/Military Facilities	Adults ≥19 to 64 with asthma	Pneumococcal vaccine education for primary/subspecialty providers with decision support via mobile app	Pneumococcal (PCV20)	Staff education
Pennant K.N., 2015 [37]	QES	USA	H	Outpatient Clinics	≥65 and high-risk patients (asthma, HIV, chronic lung disease, and immunocompromised)	Three strategies for quality improvement: physician reminders, patient letters, and a nurse-driven model	Influenza, Pneumococcal	Multi-component strategies
Poulikakos D., 2022 [66]	COH	United Kingdom	H	Tertiary/University Hospital	Adults on RRT	Online multi-disciplinary meetings to identify eligible patients, address hesitancy, and streamline vaccine delivery	COVID-19	Multi-component strategies
Rivière P., 2023 [67]	QES	France	H	Tertiary/University Hospital	Adults ≥18 undergoing oncological treatment	Staff training and standardized vaccination assessment among oncology patients	Pneumococcal, Influenza, DTP	Multi-component strategies
Runyo F., 2021 [68]	COH	France	H	Tertiary/University Hospital	Adult kidney allograft candidates	Catch-up vaccination post-ID consult, administered by dialysis staff or GP	Pneumococcal, Tdap, Influenza, HBV, HAV	Hospital-based catch-up strategy
Shafer R., 2021 [69]	COH	USA (New York)	H	Outpatient Clinics	Adults <65 with chronic conditions	Three-pronged strategy: clinician webinars, nurse-led pre-visit counseling, and interdisciplinary team huddles	Pneumococcal	Multi-component strategies
Sheth H., 2021 [70]	QES	USA (Pennsylvania)	H	Outpatient Clinics	Adults with rheumatic diseases	EMR-based Best Practice Alert system with workflow adjustments, education, and quarterly feedback to providers	PCV13, PPSV23	Multi-component strategies
Sheth H., 2017 [71]	QES	USA (Pennsylvania)	H	Outpatient Clinics	Adults ≥60 with RA	EMR-based Best Practice Alert system with workflow adjustments, education, and periodic feedback to providers	HZ	Multi-component strategies
Sitte J., 2019 [72]	COH	France	H	Outpatient Clinics	Adults with gastrointestinal cancer or IBD	Three-phase vaccination program: patient survey, ID consultation, and post-intervention knowledge reassessment	Pneumococcal, Influenza	Patient education
Tan H., 2021 [73]	QI	Singapore	H	Tertiary/University Hospital	Kidney failure adults in peritoneal dialysis	Plan–Do–Study–Act cycles: non-traditional vaccination sites, physician audit/feedback, and reminder system enhancement	Influenza, PCV13, PPSV23	Multi-component strategies
Tan L., 2020 [74]	QES	USA (Minnesota)	H	Outpatient Clinics	Adults ≥65 with chronic conditions	Evaluation of vaccine uptake after 1 year implementation of standing order protocols across 5 clinics	Tdap, PCV13, PPSV23, HZ	Standing order protocols (SOP)
Tubiana S., 2021 [75]	RCT	France and Monaco	H	General Inpatient Wards	Adults ≥65	Structured vs unstructured vaccine information and follow-up text reminders for ED patients; uptake assessed after 6 months	Influenza, Pneumococcal	Multi-component strategies
Tubiana S., 2020 [76]	CCS	France	H	Multicenter or National Studies	Adults ≥18, hospitalized for at least 24h	Structured face-to-face questionnaire administered before and after results (in person or by phone)	Influenza	Multi-component strategies
Veronese N., 2024 [77]	CSS	Italy	H	Tertiary/University Hospital	Frail adults ≥60 outpatients mainly affected by cognitive or endocrinological conditions	Free influenza/pneumococcal vaccination for frail elderly, with brochures, posters, and communication in outpatient clinics	Influenza, Pneumococcal	Multi-component strategies
Yeo Y., 2020 [78]	COH	Singapore	H	Tertiary/University Hospital	Adults ≥21 with solid organ transplant	One-stop influenza vaccination service at transplant outpatient center with pre-implementation patient survey	Influenza	Patient education

ACIP: Advisory Committee on Immunization Practices; BT: Biological Therapy; CCS: Case–Control Study; CKD: Chronic Kidney Disease; COH: Cohort Study; COPD: Chronic Obstructive Pulmonary Disease; CSS: Cross-Sectional Study; DTaP-IPV: Diphtheria, Tetanus, acellular Pertussis, Inactivated Poliovirus; DTP: Diphtheria, Tetanus, Pertussis; ED: Emergency Department; EHR: Electronic Health Record; EMR: Electronic Medical Record; GP: General Practitioner; HAV: Hepatitis A; HBV: Hepatitis B; Hib: Hemophilus influenzae type b; HIV: Human Immunodeficiency Virus; HPV: Human Papillomavirus; HZ: Herpes Zoster; H: High; IBD: Inflammatory Bowel Disease; ID: Infectious Diseases; ITP: Immune Thrombocytopenia; LT: Liver Transplant; L-M: Lower-Middle; MMR: Measles, Mumps, Rubella; PCV13: Pneumococcal Conjugate Vaccine 13-valent; PCV20: 20-valent Pneumococcal Conjugate Vaccine; PCP: Primary Care Provider; PLWH: People Living With HIV; PPSV23: Pneumococcal Polysaccharide Vaccine 23-valent; QES: Quasi-Experimental Study; QI: Quality Improvement Study; RA: Rheumatoid Arthritis; RCT: Randomized Controlled Trial; RRT: Renal Replacement Therapy; TB: Tuberculosis; Tdap: Tetanus, diphtheria, acellular Pertussis; TTP: Thrombotic Thrombocytopenic Purpura; U-M: Upper-Middle.

**Table 2:** Summary of vaccination outcomes before and after intervention, including effect size and relative increase.

First Author, Year	Total Sample Size	Age Mean	Female (%)	Total Vaccinated	Vaccinated Pre vs Vaccinated Post Intervention	Effect Measurement and Outcome	OR (95% CI)	Relative Increase (%)	Statistical Significance
Baker D.W., 2016 [35]	1255	56.8 ± 14.5	83.5%	NA	Influenza (*n* = 102) 81(79.4%) → 79 (78.2%); Pneumococcal (*n* = 1255) 360 (28.7%) → 575 (45.8%); HZ (*n* = 1255) 32 (2.5%) → 57 (4.5%)	Change in vaccination rate (pre vs. post intervention)	NA	Influenza −1.5%; Pneumococcal +59.6%; HZ +80.0%	Pneumococcal *p* < 0.0001; HZ *p* = 0.01
Bernasko N., 2023 [38]	200	Pre 40.64 ± 13.93; Post 39.92 ± 15.04	Pre 55.0%; Post 54.0%	Influenza 68; Pneumococcal 64; HBV 91; TB 93	Influenza 31.0% → 68.0%; PCV13 41.0% → 67.0%; PPSV23 41.0% → 67.0%; HBV 60.0% → 97.0%; TB 12.5%→ 99.0%	Change in vaccination rate (pre vs. post intervention)	NA	Influenza +119.0%; PCV13 +63.0%; PPSV23 +63.0%; HBV +61.7%; TB +692.0%	*p* < 0.001
Blanchi S., 2020 [39]	157 (Intervention 73; Control 84)	Intervention 78.1; Control 81.4	Intervention 65.8%; Control 55.9%	Intervention 59; Control 34	Intervention 56.2% → 80.8%; Control 38.1% → 40.5%	Comparison between intervention and control groups	NA	Intervention +43.8%; Control +6.3%	*p* < 0.001
Bock A., 2016 [40]	137 (Pre 81; Post 56)	NA	NA	Pre 2; Post 23	2.5% (2/81) → 42.0% (23/56)	Change in vaccination rate (pre vs. post intervention)	NA	+1580.0%	*p* < 0.001
Burka A., 2019 [41]	540 (Pre 270; Post 270)	Pre 71.7; Post 72.7	Pre 96.3%; Post 95.9%	Pre 9; Post 41	Any Pneumococcal 3.3% → 15.2%; PCV13 3.3% → 7.8%; PPSV23 0% → 7.4%	Change in vaccination rate (pre vs. post intervention)	NA	Any Pneumococcal +360.6%; PCV13 +136.4%; PPSV23 NA	Any Pneumococcal *p* < 0.0001; PCV 13 *p* = 0.0223; PPSV23 *p* < 0.0001
Burns C., 2018 [42]	99	NA	NA	38	0% → 38.4% within 180 days	Change in vaccination rate (pre vs. post intervention)	NA	NA	*p* < 0.001
Calmels A., 2023 [43]	39 (Reinforced 19, Standard 20)	63	69.0%	Reinforced 10; Standard 4	Reinforced 3/19 (15.8%) → 10/19 (52.6%); Standard 2/20 (10.0%) → 4/20 (20.0%); Pneumococcal: 43.6% (6/19) → 73.7% (14/19)	Change in vaccination rate (pre vs. post intervention)	NA	Reinforced +232.9%; Standard +100.0%; Pneumococcal +69.0%	Pneumococcal *p* = 0.034
Chadwick D., 2018 [44]	73	48	22.0%	HAV 53 (73.0%); HBV 63 (86.0%); Influenza 70 (96.0%); Pneumococcal 37 (51.0%)	HAV 288/520 (55.0%) → 53/73 (73.0%); HBV 273/520 (52.0%) → 63/73 (86.0%); Pneumococcal 81/520 (16.0%) → 37/73 (51.0%); Influenza 372/520 (72.0%) → 70/73 (96.0%)	Change in vaccination rate (pre vs. post intervention)	NA	HAV +32.7%; HBV +65.4%; Pneumococcal +218.8%; Influenza +33.3%	*p* < 0.01
Chan S., 2015 [45]	2517 (Intervention 1251, Control 1266)	74.5	NA	Intervention 716 (57.0%); Control 609 (48.0%)	Intervention 48% → 57%; Control 44% → 48%	Change in vaccination rate (pre vs. post intervention)	NA	Intervention +18.8%; Control +9.1%	*p* = 0.01
Coenen S., 2017 [46]	505	44 (22–70)	47.0%	159 previously vaccinated; Group A (206), Group B (140)	Influenza 10.0% → 36.0%; Pneumococcal 23.0% → 62.0%; HBV 5% → 27%; Tetanus 2.0% → 33.0%	Change in vaccination rate (pre vs. post intervention)	NA	Influenza +260.0%; Pneumococcal +169.6%; HBV +440.0%; Tetanus +1550.0%	*p* < 0.001
De Guzman E., 2022 [47]	139	NA	NA	14	10.7% → 15.4%	Change in vaccination rate (pre vs. post intervention)	NA	+43.9%	NA
Dehnen D., 2019 [48]	401	52.3	42.90%	NA	Pneumococcal 46.4% → 60.1%; Diphtheria 65.1% → 75.3%; Tetanus 88.3% → 92.8%; HBV 49.9% → 66.1%; HAV 37.4% → 48.9%; Influenza 28.9% → 23.2%	Change in vaccination rate (pre vs. post intervention)	NA	Pneumococcal +29.5%; Diphtheria +15.7%; Tetanus +5.1%; HBV +32.5%; HAV +30.7%; Influenza −19.7%	NA
Ekin T., 2023 [49]	1808 (1709 follow-up)	61.9 ± 12.1	44.6%	1122	65.3% post-recommendation (*n* = 1116)	Factors associated with vaccination	Female 1.55 (1.25–1.92); Higher education (primary vs. none) 1.57 (1.20–2.07); Knowledge 1.93 (1.56–2.40); Physician recommendation 5.12 (1.92–13.68)	NA	*p* = 0.001
Fernández-Cañabate E., 2020 [50]	188	52.5 ± 13.2	50.50%	82	43.6% (campaign flu 2016/17) → 84.0% (campaign flu 2017/18)	Change in vaccination rate (pre vs. post intervention)	NA	+92.7%	*p* = 0.636 difference between campaign 2016/17 and 2017/18; *p* < 0.001 patient vaccinated during 2017/2018
Figueroa-Parra G., 2021 [51]	Pre 73; Post 102	Pre 50.9 ± 12.4; Post 51.3 ± 14.7	Pre 94.5%; Post 82.4%	NA	Influenza 43.0% → 55.0%; Pneumococcal 26.0% → 30.0%, HZ 0% → 4.0%, HPV 4.0% → 10.0%, HBV 19.0% → 25.0%	Change in vaccination rate (pre vs. post intervention)	NA	Influenza +27.9%; Pneumococcal +15.4%; HZ NA; HPV +150.0%; HBV +31.6%	Influenza *p* = 0.118; Pneumococcal *p* = 0.563; HZ *p* = 0.084; HPV *p* = 0.138; HBV *p* = 0.350
Fujita A., 2024 [52]	1562 (Pre 769; Post 793)	NA	NA	77	16 (21%) → 61 (79%)	Change in vaccination rate (pre vs. post intervention)	Pre 1.0; post 3.92 (2.24–6.87)	+276.2%	*p* < 0.001
Guerra G.L., 2023 [36]	139	59.17 ± 12.91	62.6%	NA	Pneumococcal 22.1% → 29.4%, HBV 29.4% → 48.5%; Tetanus 51.5% → 72.1%; Influenza 79.4 → 89.7%	Comparison between intervention and control groups	NA	Pneumococcal +33.0%; HBV +65.0%; Tetanus +40.0%; Influenza +13.0%	Intervention: Influenza *p* = 0.016, HBV *p* = 0.002, Tetanus *p* = 0.007, Pneumococcal *p* = 0.049; Control: Influenza *p* = 0.302, HBV *p* = 0.122, Tetanus *p* = 0.864, Pneumococcal *p* = 0.648
Hill J.D., 2017 [53]	Influenza 145; Pneumococcal 145	NA	NA	Influenza 117; Pneumococcal 120	Influenza 72.2% → 92.9%; Pneumococcal 81.3% → 84.3%	Change in vaccination rate (pre vs. post intervention)	NA	Influenza +28.7%; Pneumococcal +3.7%	Influenza *p* = 0.001; Pneumococcal *p* = 0.638
Hooper K., 2023 [54]	142 admissions (some patients admitted more than once)	36.5	64.8%	19	Eligible during admission 67 (47.2%); Offered vaccine 45 (67.2% of eligible); Accepted offer 26 (57.8% of offered); Vaccinated 19 (73.1% of accepted; 28.3% of eligible)	Comparison between intervention and general population	NA	NA	NA
Hussain N., 2021 [55]	356 (Clinic A 174; Clinic B 182)	Clinic A 44.5; Clinic B 44.1	51.7%	Influenza: Clinic A 67.8%, Clinic B 47.8%; Pneumococcus: Clinic A 65.9%, Clinic B 62.6%; HZ: Clinic A 47.1%, Clinic B 31.1%; HAV: Clinic A 36.2%, Clinic B 22.5%; HBV: Clinic A 62.1%, Clinic B 56.7%; Tdap: Clinic A 51.7%, Clinic B 52.8%; HPV: Clinic A 56.5%, Clinic B 76.2%	Influenza 67.8% → 47.8%; Pneumococcal 65.9% → 62.6%; HZ 47.1% → 31.1%; HAV 36.2% → 22.5%; HBV 62.1% → 56.7%; Tdap 51.7% → 52.8%; HPV 56.5% → 76.2%	Change in vaccination rate (pre vs. post intervention)	NA	Influenza −29.5%; Pneumococcal –5.0%; HZ −34.0%; HAV −37.8%; HBV −8.7%; Tdap +2.1%; HPV +34.9%	Influenza *p* < 0.001; HAV *p* = 0.005; HZ *p* < 0.001; HBV *p* = 0.002; Tdap *p* < 0.001
Karakurt Z., 2024 [56]	29,530	Only age group ≥65 indicated	NA	12,187	4441 (15.0%) → 7746 (26.2%)	Change in vaccination rate (pre vs. post intervention)	NA	+74.7%	*p* < 0.001
Lee Y., 2024 [57]	3500	NA	57.0%	1318	NA	Change in vaccination rate (pre vs. post intervention)	NA	National increase +4.0%, Local increase +38.0% after SMS reminders	*p* < 0.001
Li A., 2019 [58]	348	72.8 ± 9.3	8.0%	Pre-intervention 166/348 (47.7%); Post-intervention 281/348 (80.7%)	47.7% → 80.7%	Factors associated with vaccination	For vaccination post vs. pre intervention 4.6 (3.3–6.5); For vaccine refusal post vs. pre intervention 0.31 (0.18–0.51)	+69.2%	*p* < 0.001
Liu C., 2025 [59]	7834 patients (Control 6603; Intervention 1231)	65.15 ± 9.2	14.1%	Control 107/6603 (1.6%) vs. Intervention 150/1231 (12.2%)	1.6% → 12.2%	Factors associated with vaccination	8.86	+662.5%	*p* < 0.01
Muñoz-Miralles R., 2022 [60]	524	NA	NA	115 Control group: 40 Intervention group: 75	NA	Comparison between intervention and control groups	2.48 (1.6–3.8)	NA	*p* < 0.001
Murray K., 2020 [61]	2017: 163; 2018: 262	NA	2017: 73.0%; 2018: 71.8%	NA	NA	Change in vaccination rate (pre vs. post intervention)	Influenza 9.01 (4.40–18.42); Pneumococcal 8.93 (4.39–18.17)	NA	Influenza *p* < 0.001; Pneumococcal *p* < 0.001
Mysore P., 2021 [62]	239	71 (62.77)	51.9%	84	51.0% → 75.0%	Vaccination uptake	NA	+47.1%	*p* = 0.03
Nguyen T., 2024 [63]	360	Pre 82.4 ± 6.9; Post 82.1 ± 7.0	Pre 66.9%; Post 58.7%	Pre-intervention 3 Post-intervention= 62	2.2% → 43.4%	Vaccination uptake	NA	+1872.7%	*p* < 0.001
O’Neill N., 2020 [64]	28	NA	NA	NA	Pneumococcal 14 → 19; Hib 11 → 18; Meningococcal 14 → 19; Influenza 11 → 16; Completely vaccinated 10 → 17	Vaccination uptake	NA	NA	NA
Pacheco C., 2024 [65]	NA	NA	NA	NA	17 (14.9%) → 31 (19.5%)	Vaccination uptake	NA	+30.9%	*p* = 0.33
Pennant K.N., 2015 [37]	NA	NA	NA	NA	Influenza 59.0% → 64.0% (allergy), 74.0% → 86.0% (ID); Pneumococcal 52.0% → 79.0% (pulmonary), 50.0% → 87.0% (rheumatology)	Change in vaccination rate (pre vs. post intervention)	NA	Influenza +8.5% (allergy), +16.2% (ID); Pneumococcal +51.9% (pulmonary), +74.0% (rheumatology)	NA
Poulikakos D., 2022 [66]	1156	58 (47–68)	37.6%	1118	NA	Vaccination uptake	NA	NA	NA
Rivière P., 2023 [67]	Pre 272; Post 156	NA	Pre 52.6%; Post 49.4%	NA	DTP 37.1% → 38.5%; Influenza 42.6% → 55.1%; Pneumococcal 11.8% → 15.4%	Vaccination uptake	NA	DTP +3.8%; Influenza +29.3%; Pneumococcal +30.5%	Pneumococcal *p* = 1; Influenza *p* = 0.08
Runyo F., 2021 [68]	467	58 (46- 66)	36.0%	465	NA	Vaccine acceptability	NA	NA	NA
Shafer R., 2021 [69]	370	Only age group <65 indicated	59.0%	NA	28.0% → 40.0%	Change in vaccination rate (pre vs. post intervention)	NA	+42.9%	NA
Sheth H., 2021 [70]	Phase I (patients with RA): Pre 2990, Post 5292; Phase II (all patients with rheumatic diseases): Pre 14,109, Post 26,717	Phase I (patients with RA): Pre 74 (65–98), Post 74.4 (65–101); Phase II (all patients with rheumatic diseases): Pre 58 (18–99.8), Post 59.5 (18–101)	Phase I (patients with RA) Pre 75.0%; Post 73.0%. Phase II (all patients with rheumatic diseases) Pre 73.6%; Post 74.0%	Phase I 3254, Phase II 20,682	NA	Change in vaccination rate (pre vs. post intervention)	Older age (≥65y) and treatment at academic center were associated with higher vaccination rates (OR 3.0 and 1.9, respectively; 2.7–3.3 and 1.7–2.1)	Phase 1: +33.5%, Phase II: +27.8%	*p* < 0.0001
Sheth H., 2017 [71]	Pre 1823; Post 1554	Pre 69 (60–101); Post 70 (60–95)	Pre 73.2%; Post 72.3%	988	184 → 804	Change in vaccination rate (pre vs. post intervention)	NA	NA	*p* < 0.0001
Sitte J., 2019 [72]	366	48.5	45.60%	89	Pneumococcal 16.1% → 85.7%	Change in vaccination rate (pre vs. post intervention)	NA	+432.3%	*p* < 0.0001
Tan H., 2021 [73]	249	NA	NA	NA	Influenza 63.0% → 86.0%; PCV13 54.0% → 85.0%; PPSV23 14.0% → 63.0%	Change in vaccination rate (pre vs. post intervention)	NA	Influenza +36.5%; PCV13 +57.4%; PPSV23 +350.0%	NA
Tan L., 2020 [74]	NA	NA	NA	NA	PPSV23 (high-risk, age 19–64): site D 10.0% → 23.0%, site E: 24.0% → 60.0%; PCV13 (high-risk, age 19–64): site D: 4.0% → 100.0%, site E: 30.0% → 41.0%; Tdap (age ≥65): site D: 33.0% → 38.0%, site E: 75.0% → 77.0%; PPSV23 (age ≥65): site C: 63.0% → 62.0%, site D: 21.0% → 26.0%, site E: 82.0% → 82.0%; PCV13 (age ≥65): site C: 84.0% → 72.0%, site D: 28.0% → 36.0%, site E: 71.0% → 75.0%; HZ (age ≥65): site E: 58.0% → 61.0%	Change in vaccination rate (pre vs. post intervention)	NA	PPSV23 (19–64) site D +130.0%, site E +150.0%; PCV13 (19–64) site D +2400.0%, site E +36.7%; Tdap (≥65) site D +15.2%, site E +2.7%; PPSV23 (≥65) site C –1.6%, site D +23.8%, site E 0.0%; PCV13 (≥65) site C –14.3%, site D +28.6%, site E +5.6%; HZ (≥65) site E +5.2%	*p* < 0.01
Tubiana S., 2021 [75]	1475 (Intervention 780, Control 695)	74 (69–82)	49.9%	Pneumococcal: Intervention 6.4%, Control 4.6%; Influenza: Intervention 52.1%, Control 40.0%	NA	Change in vaccination rate (pre vs. post intervention)	Pneumococcal 1.41 (0.84–2.37); Influenza: 1.63 (1.10–2.42)	NA	Pneumococcal *p* = 0.19; Influenza *p* = 0.01
Tubiana S., 2020 [76]	309	61 (45–74)	48.20%	201	65.1% → 70.4%	Change in vaccination rate (pre vs. post intervention)	NA	+8.1%	*p* = 0.02 (perception of severity); *p* = 0.49 (willingness to be vaccinated in the post-virological result questionnaire)
Veronese N., 2024 [77]	300	76.1 (60–96)	69.7%	76	NA	Change in vaccination rate (pre vs. post intervention)	NA	Influenza +19.5%; Pneumococcal +89.5%	NA
Yeo Y., 2020 [78]	308	55.3 ± 10.9	45.80%	77	77 (25.0%) → 140 (60.6%)	Change in vaccination rate (pre vs. post intervention)	0.48 (0.23–0.94) for history of diabetes and 0.95 (0.91–0.99) for a shorter number of years post transplant	+142.4%	*p* < 0.05

CI: Confidence Interval; DTP: Diphtheria, Tetanus, Pertussis; HAV: Hepatitis A; HBV: Hepatitis B; Hib: Hemophilus influenzae type b; HPV: Human Papillomavirus; HZ: Herpes Zoster; ID: Infectious Diseases; NA: Not Available; OR: Odds Ratio; PCV13: Pneumococcal Conjugate Vaccine 13-valent; PPSV23: Pneumococcal Polysaccharide Vaccine 23-valent; RA: Rheumatoid Arthritis; TB: Tuberculosis; Tdap: Tetanus, diphtheria, acellular Pertussis.

**Table 3:** Risk of bias assessment of the included studies, according to the methodological checklist used in each study (QI-MQCS, NOS, RoB 2 tool, or ROBINS-I).

First Author, Year	Checklist	Risk of Bias
Baker D.W., 2016 [35]	QI-MQCS	13
Bernasko N., 2023 [38]	NOS	9
Blanchi S., 2020 [39]	RoB 2 tool	Some concerns
Bock A., 2016 [40]	QI-MQCS	15
Burka A., 2019 [41]	NOS	8
Burns C., 2018 [42]	NOS	8
Calmels A., 2023 [43]	RoB 2 tool	Low risk
Chadwick D., 2018 [44]	ROBINS-I tool	Serious
Chan S., 2015 [45]	RoB 2 tool	Low risk
Coenen S., 2017 [46]	RoB 2 tool	High risk
De Guzman E., 2022 [47]	QI-MQCS	12
Dehnen D., 2019 [48]	NOS	6
Ekin T., 2023 [49]	NOS	7
Fernández-Cañabate E., 2020 [50]	ROBINS-I tool	Moderate
Figueroa-Parra G., 2021 [51]	QI-MQCS	14
Fujita A., 2024 [52]	ROBINS-I tool	Moderate
Guerra G.L., 2023 [36]	RoB 2 tool	High risk
Hill J.D., 2017 [53]	ROBINS-I tool	Serious
Hooper K., 2023 [54]	NOS	6
Hussain N., 2021 [55]	NOS	8
Karakurt Z., 2024 [56]	NOS	9
Lee Y., 2024 [57]	NOS	8
Li A., 2019 [58]	QI-MQCS	16
Liu C., 2025 [59]	NOS	8
Muñoz-Miralles R., 2022 [60]	RoB 2 tool	Low risk
Murray K., 2020 [61]	QI-MQCS	14
Mysore P., 2021 [62]	QI-MQCS	15
Nguyen T., 2024 [63]	ROBINS-I tool	Moderate
O’Neill N., 2020 [64]	QI-MQCS	16
Pacheco C., 2024 [65]	ROBINS-I tool	Critical
Pennant K.N., 2015 [37]	QI-MQCS	14
Poulikakos D., 2022 [66]	NOS	8
Rivière P., 2023 [67]	ROBINS-I tool	Serious
Runyo F., 2021 [68]	NOS	8
Shafer R., 2021 [69]	NOS	8
Sheth H., 2021 [70]	QI-MQCS	14
Sheth H., 2017 [71]	QI-MQCS	15
Sitte J., 2019 [72]	NOS	8
Tan H., 2021 [73]	QI-MQCS	15
Tan L., 2020 [74]	ROBINS-I tool	Serious
Tubiana S., 2021 [75]	RoB 2 tool	Some concerns
Tubiana S., 2020 [76]	NOS	8
Veronese N., 2024 [77]	NOS	8
Yeo Y., 2020 [78]	NOS	8

Color coding indicates the overall risk of bias rating: green = low risk or high quality, yellow = moderate risk, orange = serious risk or some concerns, red = high/critical risk or low quality.

## Data Availability

All data are presented in the current manuscript (text, tables, figures).

## Supplementary Materials

The following supporting information can be downloaded at: https://www.mdpi.com/article/10.3390/healthcare13141667/s1, Table S1: Literature search strategy (PubMed); Table S2: Literature search strategy (Scopus and Embase); Table S3: Inclusion/exclusion criteria based on PICOS (Population, Intervention, Comparison, Outcome, and Study Design); Table S4: Risk of Bias checklist NOS [38,41,42,48,49,54,55,56,57,59,66,68,69,72,76,77,78]; Table S5: Risk of Bias checklist QI-MQCS [35,37,40,47,51,58,61,62,64,70,71,73]; Table S6: Risk of Bias checklist RoB 2 tool [36,39,41,45,46,60,75]; Table S7: Risk of Bias checklist ROBINS-I tool [44,50,52,53,63,65,67,74]; Figure S1: (a) forest plot and (b) funnel plot of the fixed effect model assessing patient education interventions [43,48,50,51,72,78]; Figure S2: (a) forest plot and (b) funnel plot of the fixed effect model assessing multi-component strategies [35,37,40,44,47,52,53,56,58,62,63,64,67,69,71,73,76].
