# Supplementary material for: Improving Vaccine Coverage Among Older Adults and High-Risk Patients: A Systematic Review and Meta-Analysis of Hospital-Based Strategies
Source: Healthcare (Basel). 2025 Jul 10;13(14):1667. doi: 10.3390/healthcare13141667 (PMC12295636; doi:10.3390/healthcare13141667)

Supplementary Table S1. *Literature search strategy (PubMed).*

SET	Key words	Field	Records retrieved (n.)
1	"Hospital-Based"	[TiAb]	
2	"Inpatient*"		
3	"Outpatient*"		
4	"Acute Care"		
5	"Intensive Care Unit"		
6	"Primary Care"		
7	"Secondary Care"		
8	"Tertiary Care"		
9	"In-Hospital"		
10	"Emergency Unit"		
11	"Ambulatory Service"		
12	"Emergency Service, Hospital"	[MeSH Terms]	
13	"Outpatient Clinics, Hospital"		
14	"Intensive Care Units"		
15	"Hospitals"		
16	"Secondary Care Centers"		
17	"Secondary Care Centers"		
18	"Tertiary Care Centers"		
19	**Sets 1-18 were combined with "OR"**		610,271
20	"Vaccination Coverage"	[MeSH Terms]	
21	"Vaccine Uptake"	[TiAb]	
22	"Vaccine Coverage"		
23	"Vaccine Adherence"		
24	"Immunization Coverage"		
25	"Vaccination Rate"		
26	"Immunization Rate"		
27	"Immunization Adherence"		
28	"Immunization Uptake"		
29	**Sets 20-28 were combined with "OR"**		16,704
30	**Sets 19 and 29 were combined with "AND"**		1,220
31	"comment"	[Publication Type]	
32	"editorial"		
33	"letter"		
34	"news"		
35	"newspaper article"		
36	"Review"		
37	"Systematic review"	[TiAb]	
38	"Systematic Reviews as Topic"	[MeSH Terms]	
39	Review Literature as Topic		

40	Meta-Analysis	**[Publication Type]**	
41	Meta-Analysis as Topic	**[MeSH Terms]**	
42	Network Meta-Analysis	**[Publication Type]**	
43	Network Meta-Analysis as Topic	**[MeSH Terms]**	
44	**Sets 31-43 were combined with “OR”**		6,296,590
45	**Sets 30 and 44 were combined with “NOT”**		1,129
25	Set 24 was limited between 1th January 2015 and 13th March 2025		

Supplementary Table S2. *Literature search strategy (Scopus and Embase).*

Database	***Search Strategy***	***Limits/Filters Applied***	***Results (n)***
SCOPUS	*(TITLE-ABS("Hospital-Based") OR TITLE-ABS(Inpatient*) OR TITLE-ABS(Outpatient*) OR TITLE-ABS("Acute Care") OR TITLE-ABS("Intensive Care Unit") OR TITLE-ABS("Primary Care") OR TITLE-ABS("Secondary Care") OR TITLE-ABS("Tertiary Care") OR TITLE-ABS("In-Hospital") OR TITLE-ABS("Emergency Unit") OR TITLE-ABS("Ambulatory Service") OR INDEXTERMS("Emergency Service, Hospital") OR INDEXTERMS("Outpatient Clinics, Hospital") OR INDEXTERMS("Intensive Care Units") OR INDEXTERMS("Hospitals") OR INDEXTERMS("Secondary Care Centers") OR INDEXTERMS("Tertiary Care Centers")) AND (TITLE-ABS("Vaccine Uptake") OR TITLE-ABS("Vaccine Coverage") OR TITLE-ABS("Vaccine Adherence") OR TITLE-ABS("Immunization Coverage") OR TITLE-ABS("Vaccination Rate") OR TITLE-ABS("Immunization Rate") OR INDEXTERMS("Vaccination Coverage"))*	*Years: 2015–2025Document type: Article (ar)Excluded: Editorials, letters, notes, reviews, systematic reviews, narrative reviews, meta-analyses, network meta-analyses*	*2,546*

EMBASE	*('hospital-based':ti,ab OR inpatient*:ti,ab OR outpatient*:ti,ab OR 'acute care':ti,ab OR 'intensive care unit':ti,ab OR 'primary care':ti,ab OR 'secondary care':ti,ab OR 'tertiary care':ti,ab OR 'in-hospital':ti,ab OR 'emergency unit':ti,ab OR 'ambulatory service':ti,ab) AND ('vaccination coverage':ti,ab OR 'vaccine uptake':ti,ab OR 'vaccine coverage':ti,ab OR 'vaccine adherence':ti,ab OR 'immunization coverage':ti,ab OR 'vaccination rate':ti,ab OR 'immunization rate':ti,ab)*	*Years: 2015–2025Limits: Humans onlyExcluded: Editorials, letters, notes, reviews, systematic/narrative reviews, meta-analyses, network meta-analyses*	*1,707*

Supplementary Table S3. *Inclusion/Exclusion criteria based on PICOS (Population, Intervention, Comparison, Outcome, Study Design).*

Search strategy	***Details***
Inclusion criteria	P: Adults aged ≥60 years or high-risk patients aged ≥18 years (e.g., with oncological diseases, immunosuppressive conditions, diabetes, cardiovascular diseases, or other chronic conditions).<br>I: Hospital-based interventions aimed at improving uptake of recommended vaccinations. May include clinical, behavioural, or structural strategies.<br>C: No intervention, standard/usual care, alternative non-hospital-based interventions, or pre-post intervention comparisons.<br>O: Primary outcome: Vaccine uptake rate (proportion of eligible individuals who receive recommended vaccinations).<br>Secondary outcomes: Barriers and facilitators to vaccine uptake, effectiveness across hospital settings and patient subgroups.<br>S: Peer-reviewed experimental and observational studies published in English, including randomized controlled trials (RCTs), quasi-experimental studies, cohort studies (prospective or retrospective), case-control studies, and pre-post intervention studies.
Exclusion criteria	P: General population not at high risk and aged <60 years. Studies not reporting data specifically for individuals aged ≥60 or high-risk patients aged 18–64.<br>I: Interventions not delivered in hospital settings (e.g., outpatient or community-based), or not aimed at increasing vaccine uptake.<br>C: No comparison, or comparisons not relevant to the impact of hospital-based interventions on vaccine uptake.

	O: Outcomes not focused on vaccine uptake (e.g., knowledge, attitudes, intentions), or outcomes not clearly measured or not reported separately for target populations.<br>S: Conference abstracts, editorials, books, systematic or narrative reviews, commentaries, expert opinions, qualitative studies, ongoing trials or non-original research, non-human studies, non-observational studies (e.g., preclinical or methodological), studies not published in English or not peer-reviewed.
Language	English
Time filter	None

Supplementary Table S4. *Risk of Bias checklist NOS [38,41,42,48,49,54-57,59,66,68,69,72,77,78,76].*

	Selection				Comparability	Exposure			Total quality score
Record (year)	exposed cohort	non exposed cohort	ascertainment of exposure	outcome was not present at start of study	comparability of cohorts	assessment of outcome	length of follow up	adequacy of follow up	
Bernasko N.,2023	1	1	1	1	2	1	1	1	9
Burka A., 2019	1	1	1	1	2	1	0	1	8
Burns C., 2018	1	1	1	1	1	1	1	0	8
Dehnen D., 2019	1	1	0	1	1	1	1	0	6
Ekin, T., 2022	1	1	0	1	2	1	0	1	7
Hooper K., 2023	1	0	1	1	1	1	0	0	6
Hussain N.,2021	1	1	1	1	2	1	1	0	8
Karakurt Z.,2024	1	1	1	1	2	1	1	1	9
Lee Y., 2024	1	1	1	1	2	1	1	0	8
Liu C., 2025	1	1	1	1	2	1	1	0	8
Poulikakos D, 2022	1	1	1	1	1	1	1	1	8
Runyo F, 2021	1	1	1	1	1	1	1	1	8
Shafer R, 2021	1	1	1	1	1	1	1	1	8
Sitte J., 2019	1	1	1	1	2	1	1	0	8
Veronese N., 2024	1	1	1	1	2	1	1	0	8
Yeo Y., 2020	1	1	1	1	2	1	1	0	8

	Selection				Comparability	Exposure			Total quality score
Record (year)	adequate case	rapresentativeness of the cases	selection of control	definition of control	comparability of case and controls	ascertainment of exposure	same method case/controls	non response rate	
Tubiana S., 2020	1	1	1	1	2	1	1	0	8

Supplementary Table S5*. Risk of Bias checklist QI-MQCS [35,40,47,51,58,62,61,64,37,71,70,73].*

	DOMAIN																
Record (Year)	1. ORGANIZATIONAL MOTIVATION	2. INTERVENTION RATIONALE	3. INTERVENTION DESCRIPTION	4. ORGANIZATIONAL CHARACTERISTICS	5. IMPLEMENTATION	6. STUDY DESIGN	7. COMPARATOR	8. DATA SOURCE	9. TIMING	10. ADHERENCE / FIDELITY	11. HEALTH OUTCOMES	12. ORGANIZATIONAL READINESS	13. PENETRATION / REACH	14. SUSTAINABILITY	15. SPREAD	16. LIMITATIONS	TOTAL
Baker DW, 2016	1	1	1	1	1	1	1	1	1	0	1	1	1	0	0	1	13
Bock A, 2016	1	1	1	1	1	1	1	1	0	1	1	1	1	1	1	1	15
De Guzman E, 2022	1	1	1	1	1	1	1	1	1	0	1	0	1	0	1	0	12
Figueroa-Parra G, 2021	1	1	1	0	1	1	0	1	1	1	1	1	1	1	1	1	14
Li A, 2019	1	1	1	1	1	1	1	1	1	1	1	1	1	1	1	1	16
Mysore P, 2021	1	1	1	1	1	1	0	1	1	1	1	1	1	1	1	1	15
Murray K, 2020	1	1	1	1	1	1	1	1	1	1	1	1	1	0	1	0	14
O'Neill N, 2020	1	1	1	1	1	1	1	1	1	1	1	1	1	1	1	1	16
Pennant KN, 2015	1	1	1	0	1	1	1	1	1	1	1	1	1	1	0	1	14
Sheth H, 2017	1	1	1	1	1	1	1	1	1	1	1	1	1	1	1	0	15
Sheth H, 2021	1	1	1	1	1	1	1	1	1	1	1	1	1	0	0	1	14
Tan H, 2021	1	1	1	1	1	1	1	1	1	0	1	1	1	1	1	1	15

Supplementary Table S6. *Risk of Bias checklist RoB 2 tool [39,41,45,46,36,60,75].*

Record (Year)	D1	D2	D3	D4	D5	Overall
Blanchi, S. (2020)	Low risk	Some concerns	Low risk	Some concerns	Low risk	Some concerns
Calmels, A. (2023)	Low risk	Some concerns	Low risk	Low risk	Low risk	Low risk
Chan, S. (2015)	Low risk	Low risk	Low risk	Low risk	Low risk	Low risk
Coenen, S. (2017)	Some concerns	High risk	Low risk	Some concerns	Low risk	High risk
Guerra, G.L. (2023)	Low risk	Some concerns	Low risk	High risk	Low risk	High risk
Muñoz-Miralles, R. (2022)	Low risk	Low risk	Low risk	Low risk	Low risk	Low risk
Tubiana, S. (2021)	Some concerns	Some concerns	Low risk	Some concerns	Low risk	Some concerns

Supplementary Table S7. *Risk of Bias checklist ROBINS-I tool [44,50,52,53,63,65,67,74].*

Record (Year)	D1	D2	D3	D4	D5	D6	D7	Overall
Chadwick, D. 2018	Serious	Moderate	Low	Moderate	Moderate	Low	Moderate	Serious
Fernández-Cañabate, E. 2020	Moderate	Moderate	Low	Low	Moderate	Moderate	Low	Moderate
Fujita, A. 2024	Moderate	Low	Low	Low	Moderate	Low	Moderate	Moderate
Hill J.D. 2017	Serious	Moderate	Low	Low	Low	Low	Moderate	Serious
Nguyen, T. 2024	Moderate	Low	Low	Moderate	Low	Low	Low	Moderate
Pacheco, C. 2024	Critical	Moderate	Low	Moderate	Serious	Serious	Moderate	Critical
Rivière, P. 2023	Serious	Moderate	Low	Moderate	Low	Low	Moderate	Serious
Tan, L. 2020	Serious	Moderate	Low	Moderate	Low	Moderate	Low	Serious

Supplementary Figure S1. (**a**) forest plot and (**b**) funnel plot of the fixed effect model assessing patient education interventions [43,48,50,51,72,78].

(a)

	ES	95% CI	Sig.	N
Calmels (pneumo) 2023	6.07	2.24 , 16.41	0.000	38
Dehnen (HAV) 2019	1.60	1.31 , 1.95	0.000	802
Dehnen (HBV) 2019	1.96	1.60 , 2.40	0.000	802
Dehnen (dipht) 2019	1.64	1.32 , 2.03	0.000	802
Dehnen (flu) 2019	0.74	0.59 , 0.93	0.009	802
Dehnen (pneumo) 2019	1.74	1.43 , 2.12	0.000	802
Dehnen (tet) 2019	1.70	1.20 , 2.41	0.003	802
Fernandez-Canabate (flu) 2020	6.81	4.79 , 9.67	0.000	376
Figueroa-Parra (HBV) 2021	1.37	0.81 , 2.31	0.243	175
Figueroa-Parra (HPV) 2021	2.54	0.93 , 6.88	0.068	175
Figueroa-Parra (HZ) 2021	6.72	0.58 , 77.22	0.126	175
Figueroa-Parra (flu) 2021	1.65	1.07 , 2.53	0.022	175
Figueroa-Parra (pneumo) 2021	1.24	0.77 , 2.00	0.375	175
Sitte (pneumo) 2019	31.42	23.60 , 41.84	0.000	732
Yeo (flu) 2021	2.50	1.96 , 3.19	0.000	616
Overall (fixed-effect model)	2.11	1.96 , 2.27	0.000	7449

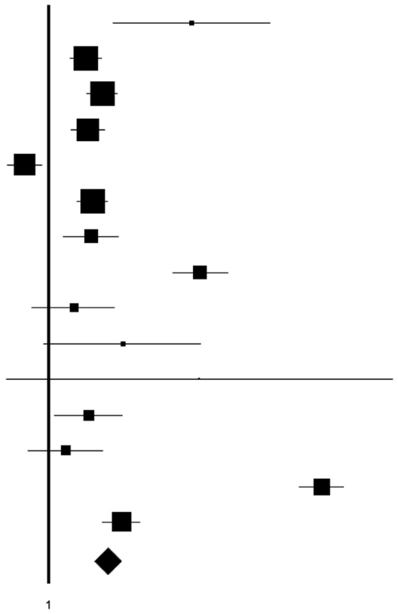

(b)

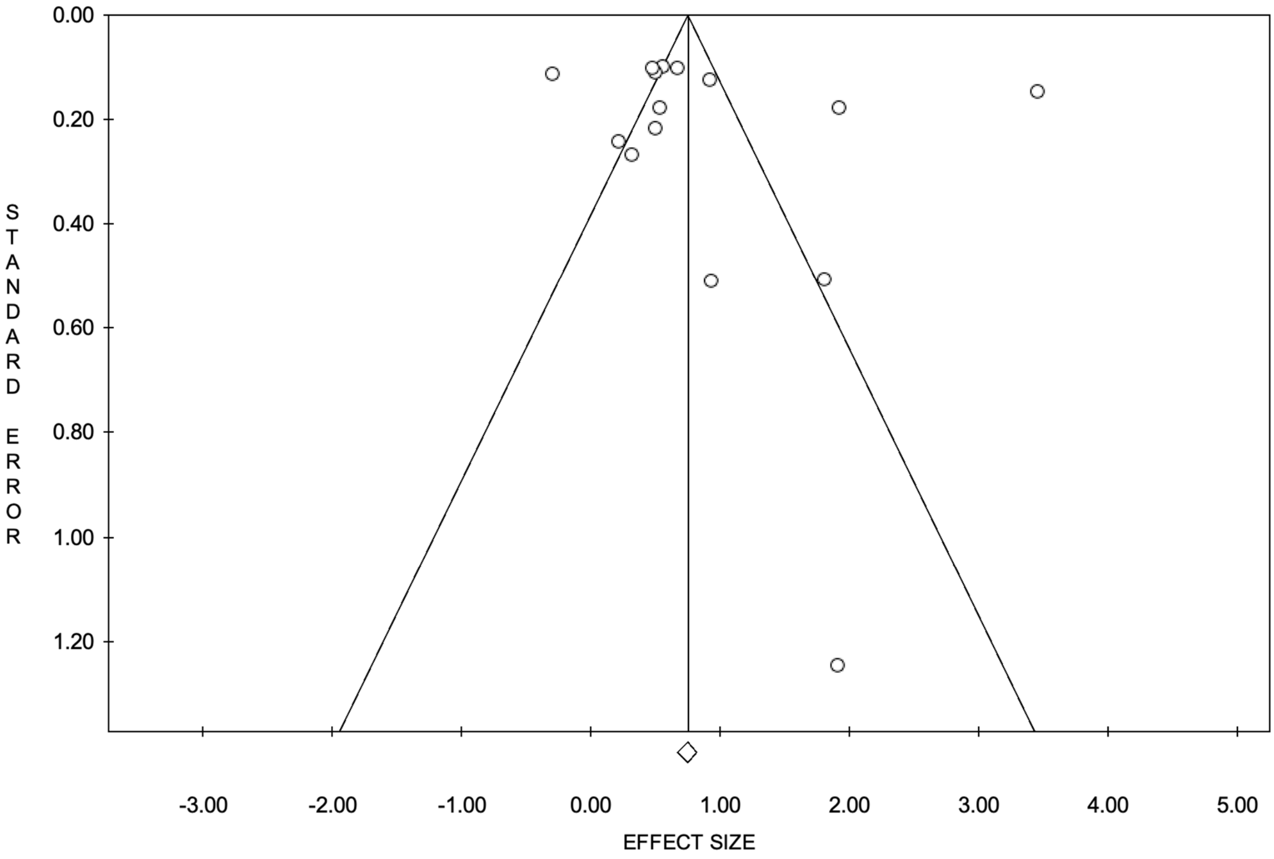

Supplementary Figure S2. (**a**) forest plot and (**b**) funnel plot of the fixed effect model assessing multi-component strategies [35,40,44,47,52,53,56,58,62-64,37,67,69,71,73,76].

(a)

	ES	95% CI	Sig.	N
Baker (HZ) 2016	1.82	1.32 , 2.50	0.000	2510
Baker (flu) 2016	0.89	0.56 , 1.43	0.630	204
Baker (pneumo) 2016	2.10	1.87 , 2.36	0.000	2510
Bock (pneumo) 2016	27.53	8.11 , 93.42	0.000	137
Chadwick (HAV) 2018	2.13	1.43 , 3.18	0.000	593
Chadwick (HBV) 2018	5.70	3.30 , 9.86	0.000	593
Chadwick (flu) 2018	9.28	3.35 , 25.70	0.000	593
Chadwick (pneumo) 2018	5.57	4.05 , 7.66	0.000	593
De Guzman (COVID-19) 2022	1.47	0.89 , 2.43	0.133	278
Fujita (COVID-19) 2024	14.54	8.38 , 25.21	0.000	154
Hill (flu) 2017	5.14	2.94 , 8.98	0.000	290
Hill (pneumo) 2017	1.21	0.79 , 1.87	0.380	290
Karakurt (pneumo) 2024	2.01	1.95 , 2.07	0.000	59060
Li (flu) 2019	4.60	3.61 , 5.86	0.000	696
Mysore (HBV) 2021	2.86	2.17 , 3.77	0.000	478
Nguyen (pneumo) 2024	33.65	18.04 , 62.75	0.000	720
O'Neill (Hib) 2020	2.78	1.29 , 5.98	0.009	56
O'Neill (flu) 2020	2.06	0.97 , 4.38	0.060	56
O'Neill (meningo) 2020	2.11	0.98 , 4.55	0.057	56
O'Neill (pneumo) 2020	2.11	0.98 , 4.55	0.057	56
Pennant (flu)a 2015	1.23	1.10 , 1.39	0.001	2284
Pennant (flu)b 2015	2.16	1.77 , 2.64	0.000	1318
Pennant (penumo)b 2015	6.69	6.08 , 7.36	0.000	5796
Pennant (pneumo)a 2015	3.48	3.18 , 3.80	0.000	4966
Riviere (DTP) 2023	1.06	0.79 , 1.41	0.699	428
Riviere (flu) 2023	1.65	1.25 , 2.19	0.000	428
Riviere (pneumo) 2023	1.36	0.92 , 2.03	0.127	428
Shafer (pneumo) 2021	1.71	1.37 , 2.12	0.000	740
Sheth (HZ) 2017	9.55	8.35 , 10.92	0.000	3377
Tan (flu) 2021	3.58	2.60 , 4.93	0.000	498
Tan (pneumo)a 2021	4.92	3.60 , 6.71	0.000	498
Tan (pneumo)b 2021	10.43	7.58 , 14.36	0.000	498
Tubiana (flu) 2020	1.29	1.01 , 1.64	0.039	618
Overall (fixed-effect model)	2.39	2.33 , 2.44	0.000	91800

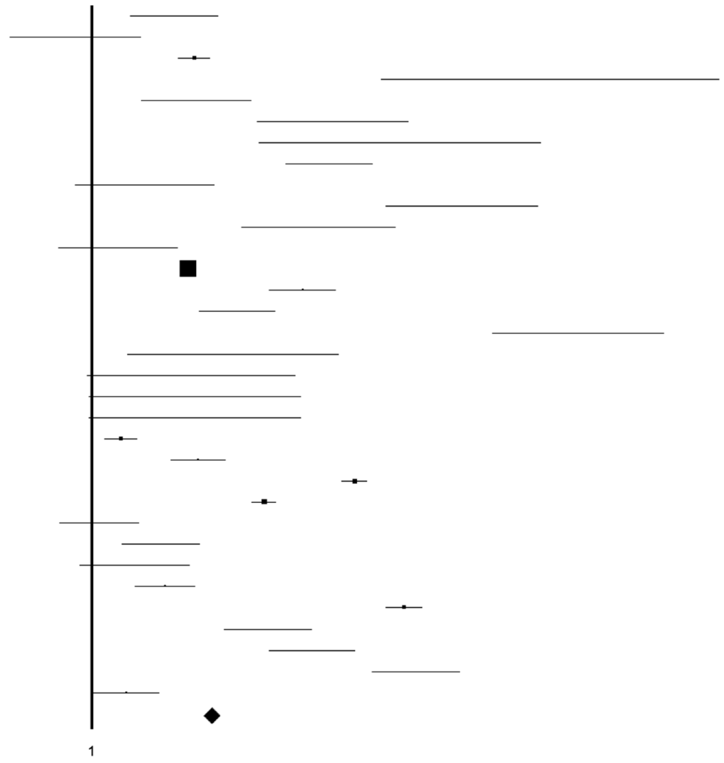

(b)

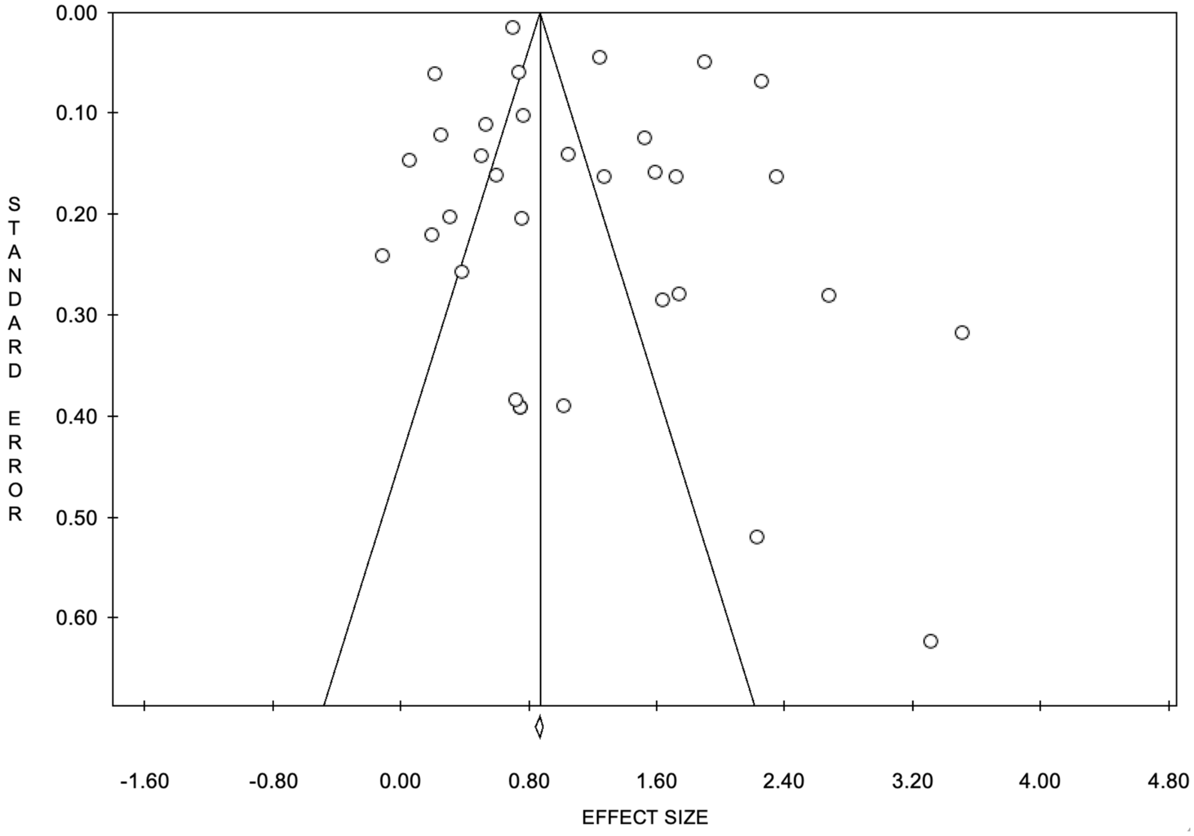

Supplement: Supplementary file 1 [file healthcare-13-01667-s001.zip › healthcare-3674383-supplementary.pdf]
